# SuPReMe: a rapid reverse genetics method to generate clonal populations of recombinant RNA viruses

**DOI:** 10.1038/s41426-018-0040-2

**Published:** 2018-03-21

**Authors:** Jean-Sélim Driouich, Souand Mohamed Ali, Abdennour Amroun, Fabien Aubry, Xavier de Lamballerie, Antoine Nougairède

**Affiliations:** UMR “Émergence des Pathologies Virales” (EPV: Aix-Marseille Univ – IRD 190 – Inserm 1207 – EHESP – IHU Méditerranée Infection), Marseille, France

## Abstract

Reverse genetics systems enable the manipulation of viral genomes and are proving to be essential for studying RNA viruses. Methods for generating clonal virus populations are particularly useful for studying the impact of genomic modifications on viral properties. Here, by exploiting a chikungunya virus model, we compare viral populations and their replicative fitness when generated using either the rapid and user-friendly PCR-based ISA (Infectious Subgenomic Amplicons) method or classical infectious clone technology. As anticipated, the ISA method resulted in greater genetic diversity of the viral populations, but no significant difference in viral fitness in vitro was observed. On the basis of these results, a new ISA-derived reverse genetics procedure was developed. This method, designated ‘SuPReMe’ (Subgenomic Plasmids Recombination Method), in which digested plasmids containing subgenomic DNA fragments were directly transfected into permissive cells, retains the following major advantages of the ISA method: it is rapid, flexible and does not require the cloning of complete genomes. Moreover, SuPReMe has been shown to produce virus populations with genetic diversity and replicative fitness similar to those obtained using conventional infectious clone technology. SuPReMe, therefore, represents an effective and promising option for the rapid generation of clonal recombinant populations of single-stranded positive-sense RNA viruses.

## Introduction

The study of RNA viruses has greatly benefited from the development of reverse genetics systems that enable the generation of infectious viruses from genomic DNA copies and facilitate the manipulation of viral genomes^1,2^. Importantly, reverse genetics enables the deciphering of RNA virus life cycles and mechanisms of pathogenesis and the development of novel antiviral compounds and new vaccine strategies^3,4^.

Several reverse genetics procedures are now available to produce wild-type and genetically modified viruses, and each procedure has inherent benefits and drawbacks^1^. If the objective is to study virus/host interactions during the natural cycle of the virus, methods that conserve the original mutant spectrum may be relevant^5^. Conversely, if the objective is to determine the impact of mutations on the biological properties of the virus, methods that generate (quasi-)clonal (i.e., very homogeneous) populations of viruses, such as infectious clone (IC) technology, are of specific interest^6^. Indeed, IC methodology remains the most widely used reverse genetics system^7^. However, despite numerous technological advances that have simplified the construction and use of IC, this method remains difficult to manipulate, particularly because of the instability and toxicity of certain viral sequences expressed in bacteria^7–9^.

This limitation resulted in the development of new bacterium-free approaches^9–12^, among which the ISA (infectious subgenomic amplicons) method has been successfully applied to a variety of single-stranded positive-sense RNA viruses^12^. This rapid procedure requires no additional step, such as cloning or in vitro transcription, to produce infectious viruses because both the assembly of subgenomic amplicons and viral RNA production occur directly in cellulo^12^.

To develop an ISA-based rapid and simple procedure for generating clonal populations of recombinant viruses, we used the chikungunya virus (CHIKV; family *Togaviridae*; genus *Alphavirus*; a small, enveloped, single-strand positive-sense RNA virus) as a model^13^. CHIKV, a human pathogen of African origin, is transmitted by *Aedes* spp. mosquitoes and is responsible for febrile arthralgia in humans^14^. In recent years,CHIKV has generated massive epidemics on islands in the Indian and Pacific Oceans, India, Southeast Asia and the Americas^14–16^.

Even when high-fidelity polymerases are used, PCR amplification can generate a low rate of undesired nucleotide changes. Therefore, PCR-based reverse genetics methods — such as the ISA method — are expected to be associated with artificial viral heterogeneity^17^. To confirm this assumption, we investigated the impact of the ISA method on the genetic diversity of viral populations in comparison with the infectious clone procedure. Because it is well established that the mutant spectrum (i.e., intra-population variability) shapes the virus phenotype (e.g., their replicative fitness)^18–20^, we also studied the impact of the ISA method on the viral phenotype in vitro.

On the basis of this initial comparative study, we developed a new ISA-derived reverse genetics method that we designated ‘SuPReMe’ (Subgenomic Plasmids Recombination Method). With this technique, we produced clonal populations of engineered viruses while retaining the advantages of the original ISA method (i.e., the use of subgenomic DNA fragments, rapidity, flexibility and versatility).

## Materials and methods

### Cells

Vero ATCC cells (derived from the kidney of an African green monkey; ATCC number CCL-81) were cultured at 37 °C with 5 % CO_2_ in minimal essential medium (Life Technologies) with 7 % heat-inactivated fetal bovine serum (FBS; Life Technologies), 1 % penicillin/streptomycin (PS; 5000 U ml^−1^ and 5000 µg ml^−1^; Life Technologies) and 1% glutamine (Gln; 200 mmol l^−1^; Life Technologies). HEK-293 cells (ATCC number CCL-1573) were cultured at 37 °C with 5 % CO_2_ in the same medium used for the Vero ATCC cells supplemented with 1 % non-essential amino acids (Life Technologies).

### CHIKV infectious clones (ICs)

We used a previously described IC (referred to as WT) of the LR2006 strain of CHIKV^21^ (GenBank accession EU224268). The complete genome of the virus is flanked at the 5′ and 3′ termini by the human cytomegalovirus promoter (pCMV) and the simian virus 40 polyadenylation signal (HDR/SV40pA), respectively. All of these regions were inserted into a modified pBR322 plasmid. We modified this IC by introducing one synonymous mutation at position 4420 (C → A; designated *WT). Briefly, the fragment of interest was removed from the coding sequence of the WT Infectious Clone (IC) by digestion (MfeI and AgeI) and replaced by the mutated fragment. The mutated fragment was obtained using two overlapping corrected primers. The two resulting PCR products were then merged by fusion PCR. The integrity of both ICs was confirmed by restriction mapping (NheI) and their sequences were verified by sequencing^21^.

### Preparation of DNA fragments for the ISA procedure

CHIKV complete genomes, flanked, respectively, at the 5′ and 3′ termini by pCMV and HDR/SV40pA, were amplified by PCR in three overlapping DNA fragments with lengths of 4.8 kb, 2.9 kb and 5.2 kb. Amplicons were produced using a Platinum PCR SuperMix High Fidelity kit (Life Technologies). The mixture (final volume, 50 µl) consisted of 45 µl of SuperMix, 2 µl of DNA template at 1 ng µl^−1^ (infectious clone) and 200 nM aliquots of each primer. Amplifications were performed on a Biometra Professional Standard Gradient thermocycler with the following conditions: 94 °C for 2 min, followed by 40 cycles of 94 °C for 15 s, 56 °C for 30 s, 68 °C for 5 min. The size of the PCR fragments was verified by gel electrophoresis, and then the PCR products were purified (Amicon Ultra 0.5 ml kit; Millipore). Because ICs were used as the template, the complete removal of the template was ensured by a digestion step using the restriction enzyme *Dpn*I (New England Biolabs) before transfection, as previously described^12^.

### Preparation of DNA fragments for the SuPReMe

Each overlapping DNA fragment used to generate the *WT virus, was cloned into a bacterial plasmid (StrataClone Vector Mix amp/kan; Agilent). All the DNA fragments were similar to those used in the ISA procedure. NotI and AvrII restriction sites were added to the 5′ and 3′ ends, respectively, of each DNA fragment before cloning. The integrity of these three plasmids was confirmed by restriction mapping and verified by sequencing. DNA fragments for the SuPReMe were generated by digesting these plasmids with NotI and AvrII restriction enzymes at 37 °C overnight. Before transfection, the digestion products were analyzed by gel electrophoresis and purified (Amicon Ultra 0.5 ml kit; Millipore).

### Cell transfection

A 1 µg aliquotof an equimolar mix of the three cDNA fragments (amplicons or digestion products) or 1 µg of an infectious clone was transfected. Two mixtures were prepared. The first mixture contained 125 μl of optiMEM (modified Eagle’s minimum essential medium buffered with HEPES and sodium bicarbonate and supplemented with hypoxanthine, thymidine, sodium pyruvate, L-glutamine, trace elements, and growth factors) (Gibco®) and 7.5 μL of Lipofectamine 3000 (a cationic liposome based reagent that provides high transfection efficiency) (Life Technologies). The second mixture contained 125 μl of optiMEM (Gibco®), 1 μg of the DNA to be transfected and 2 µl of P3000 Enhancer Reagent (Life Technologies). After the two mixtures were incubated for 5 min at room temperature, they were mixed together and then left for another 5 min at ambient temperature. This final mixture was added to a 12.5 cm^2^ culture flask containing subconfluent HEK-293 cells and 2 ml of medium without antibiotics. After 24 h at 37 °C, the cell supernatant medium was removed, the cells were washed twice in Hank’s balanced salt solution (HBSS; Life Technologies), and 3.5 ml of fresh medium was added. The cell supernatant medium was harvested after 6 to 7 days, clarified by centrifugation, aliquoted and stored at −80 °C. Each virus was then passaged once inVero ATCC cells. Passages were executed by inoculating 333 µl of clarified infectious supernatant medium onto cells in a 12.5 cm^2^ culture flask containing 666 µl of medium. After incubation for 2 h at 37 °C in 5% CO_2_, the cells were washed twice with HBSS, and 3.5 ml of fresh medium was added. Then, the supernatant medium was collected and aliquoted as described above. The resulting virus stocks were used as the source for quantification of viral RNA, TCID_50_ assay, virus competition experiments and whole-genome sequencing.

### Quantitative real-time PCR assays

To assess the production of viral particles in the cell supernatant medium, viral RNA yields were quantified using the GoTaq Probe 1-Step RT-qPCR System (Promega), and the remaining DNA was detected usinga Takyon kit (Eurogentec). AnEZ1 mini virus 2.0 kit and anEZ1 Biorobot (both from Qiagen) were used to extract RNA according to the manufacturer’s instructions. The GoTaq mixture (final volume: 20 µl) contained 10 µl of 2 × Master Mix, 0.5 µl of each primer (10 µM), 0.13 µl of specific probe (10 µM), 0.5 µl of GoSCRIPT RT Mix, 0.87 µl of nuclease-free water and 7.5 µl of extracted nucleic acids. The Takyon mixture (final volume: 20 µl) comprised 10 µl of Takyon TM Master Mix, 0.5 µl of each primer (10 µM), 0.16 µl of specific probe (10 µM), 3.84 µl of nuclease-free water and 5 µl of extracted nucleic acids. Assays were performed using the CFX96 Touch real-time PCR Detection System (Bio-Rad) with the following conditions: 50 °C for 15 min, 95 °C for 2 min, followed by 40 cycles of 95 °C for 15 s, 60 °C for 1 min. The data collection occurred during the 60 °C step. The amount of DNA was negligible compared to the RNA thresholds (10^6^ times lower). The difference between the cycle threshold values (*C*_t_) obtained by real-time PCR assays was used to assess viral RNA production. Yields of viral RNA, expressed as dose detection limits (arbitrary unit; AU), were assessed from standard curves (standards comprised viral RNA extracted from the supernatant media of cultured viruses).

### Tissue-culture infectious dose 50 (TCID_50_) assay

For each determination of TCID_50_, a 96-well culture plate containing confluent Vero ATCC cells with 100 µl of medium per well was inoculated with 50 μl per well of serial 10-fold dilutions of clarified cell supernatant medium. Each row included six wells of the same viral dilution and two negative controls. The plates were incubated for 7 days and then read for the absence or presence of cytopathic effect (CPE) in each well. The determination of the TCID_50_ ml^−1^ was performed using the method described by Reed and Muench^22^.

### In cellulo competition experiments

To compare the replicative fitnesses of the viruses generated using either the IC or the ISA procedure, the WT virus was competed with the *WT virus. Each virus was generated using both methods in duplicate (two independent transfections). The WT virus produced from the IC procedure was competed with the *WT virus produced using the ISA method (two competitions with viruses from two transfections), and the *WT virus produced from the IC procedure was competed with the WT virus produced using the ISA method (two competitions with viruses from two transfections). For each of these four competition experiments, three wells of a 6-well culture plate containing confluent Vero ATCC cells were inoculated with a mix of both viruses (TCID_50_ ratio of 50/50; global multiplicity of infection of 0.5) for 2 h at 37 °C with 5% CO_2_. Then, the plates were washed (HBSS) and incubated for 48 h after the addition of 4 ml of fresh medium. The recovered infectious cell supernatant medium was then passaged 10 times in the same manner (6-well culture plate of Vero ATCC cells). Viral RNA was extracted from 200 µl of clarified cell supernatant media using an EZ1 mini virus 2.0 kit and an EZ1 Biorobot (both from Qiagen). The relative proportion of each virus was evaluated by amplifying and sequencing a genomic fragment of 252 bp encompassing position 4420, which differentiates the viruses (see above). The following primers were used with a Superscript III One-Step RT-PCR Platinum *Taq* High Fidelity kit (Life Technologies): forward, 5′-ACTTCTCTAATTATTCGGAGTCT-3′, at position 4291 and reverse, 5′-TGGCCTCAGATATTTTCTTCTC-3′, at position 4521. The amplicons were sequenced as described below (Sequence analysis of the full-length genome), and the proportion of bases C/A at position 4420 was used to calculate the relative amount of each virus.

### Sequence analysis of the full-length genome

The cell supernatant media were clarified and treated with viral lysis buffer AVL (Qiagen). Following RNA extraction using an EZ1 mini virus 2.0 kit and an EZ1 Biorobot (both from Qiagen), a set of specific primer pairs was used to generate overlapping amplicons covering the full-length genome (excluding the 41 nt upstream polyA tail) with the RT-PCR Taq HIFI System (Invitrogen). The amplified DNA was analyzed using an Ion PGM Sequencer^23^ (Life Technologies) to perform complete genome sequencing. The read sequences obtained were analyzed with CLC Genomics Workbench 6 software. They were trimmed, first using the quality score, then by removing the primers used during the amplification and finally at the 5′ and 3′ termini by systematically removing 6 nucleotides. Reads with a length greater than 29 nucleotides were used and mapped to the original genome sequence, which was used as a reference. To assess the intra-population genetic diversity, the mutation frequencies for each position were calculated as the number of reads with a mutation compared to the reference divided by the total number of reads at that site. Only substitutions with a frequency of at least 1% were taken into account for the analysis (Supplementary Table S1).

### Adaptation experiment

A previously described re-encoded CHIKV was used^21^. This virus was transfected as previously described and then passaged five times. At each passage, the cell supernatant media were clarified and extracted as previously described. Viral genomes from the supernatant media were sequenced using the Ion PGM Sequencer^23^ (Life Technologies). The intra-population genetic diversity was assessed as previously described. In addition, infectivity assays (TCID_50_/ml) were performed on the supernatant media before the first passage and at the first and fourth passages.

### Statistical analysis

All the tests were carried out with R software^24^. This includes the Shapiro–Wilk test, Wilcoxon test, Fischer test, Welch *t* test and Student *t* test.

## Results

### Comparison of viruses generated using either an infectious clone or the ISA method

#### Production of viruses

Infectious clones were derived from CHIKV strain LR2006 OPY, hereafter referred to as the WT (wild-type) virus. The original IC construct^21^, WT_IC, was used for the production of the infectious WT_IC virus. A slightly different IC, the *WT_IC construct, which was differentiated by one synonymous mutation (marker C → A at position 4420), was used for production of the infectious *WT_IC virus. Using these ICs as PCR templates, viruses were also produced using the ISA method (designated WT_ISA virus and *WT_ISA virus, respectively).

For all constructs, nucleic acids were transfected into HEK-293 cells. All infectious viruses were successfully recovered and passaged once in Vero ATCC cells. Stocks of infectious cell supernatant media were used for further analyses.

#### Evaluation of genetic diversity

To evaluate the impact of the reverse genetics method on the genetic diversity of viral populations, the complete genome sequences of WT_IC, WT_ISA, *WT_IC and *WT_ISA viruses were established in triplicate by next-generation sequencing (using the sequences of the ICs as a reference and including substitutions with a frequency ≥1% in the mutant analyses).

**Fig. 1:**
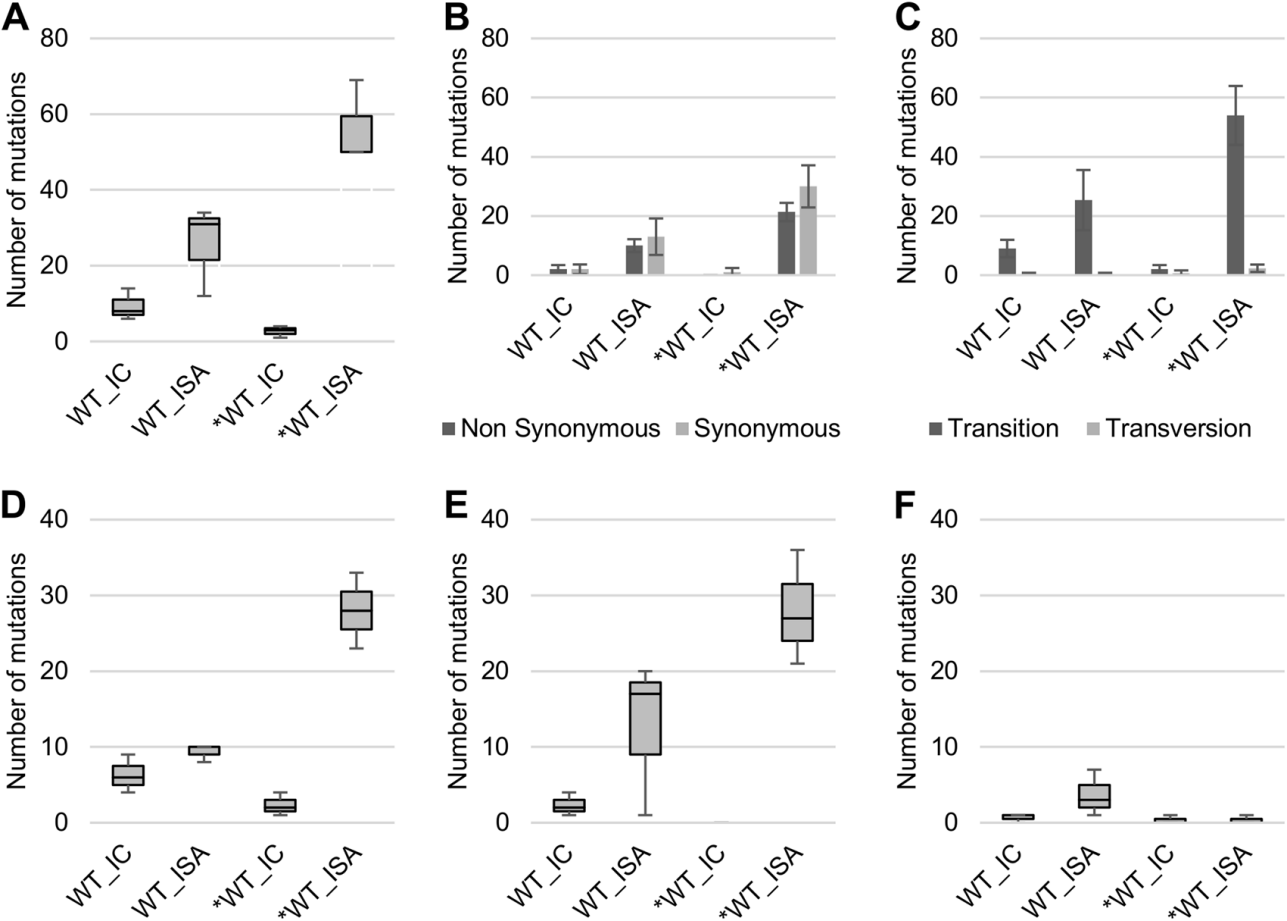
Impact of the ISA method on genetic diversity of viral populations. To investigate the impact of reverse genetics on viral population heterogeneity, the complete genome sequence of each virus was determined in triplicate. **a** Represents the number of mutations detected. Mutation characteristics are represented in **b** (Nonsynonymous/Synonymous mutations) and **c** (Transition/Transversion). **d**–**f** Represent the number of low-, mid- and high-frequency mutations, respectively. In **a**, **d**–**f**, the bottom and top of the box represent the first and third quartiles, the band inside the box represents the median value and the ends of the error bars represent the minimum and maximum values. In **b**, **c**, the average number of mutations is shown, and the error bars represent the standard deviation

Viruses generated using the ISA method exhibited a higher average number of mutations than viruses produced using ICs (25.67 vs. 9.33 and 56.33 vs. 2.67 for WT and *WT viruses, respectively; Fig. 1a). This difference was significant for the *WT virus (Welch *t* test; *p* = 0.0124).

Regardless of the reverse genetics method used to generate the virus, we observed a majority of transitions and no significant differences in the proportion of synonymous and non-synonymous mutations (Fig. 1b, c).Mutations detected in viruses produced using the ISA method were distributed randomly throughout the whole genome, which is in line with the expected distribution pattern of PCR errors. In contrast, mutations detected in viruses produced using ICs were primarily located in the 5′ UnTranslated Region (UTR) and the nsP1 region (70 and 86% of the mutations sites for the WT and *WT viruses, respectively; Supplementary Figure S1).

Mutations were assigned to three categories according to their frequencies (calculated for each position as the number of reads with a mutation divided by the total number of reads) (Supplementary Figure S2), i.e., low-frequency (<6% of reads), mid-frequency (6–30%) and high-frequency mutations (>30%). The average number of low- and mid-frequency mutations was higher when viruses were produced using the ISA method (Fig. 1d, e). This difference was significant only for the *WT virus (Student *t* test; *p* = 0.008 for low-frequency mutations). The average number of high-frequency mutations was extremely low, except for the WT_ISA viruses (Fig. 1f). This difference was primarily due to the high number of high-frequency mutations (*n* = 7) detected for the WT_ISA #3 virus.

Finally, the proportion of mutations shared between triplicates of the same virus was higher for viruses produced from ICs (Supplementary Figure S3), suggesting that ISA mutations were primarily randomly determined, while IC mutations may partly reflect early adaptation events to propagation in cell cultures.

#### Evaluation of replicative fitness in cellulo

**Fig. 2:**
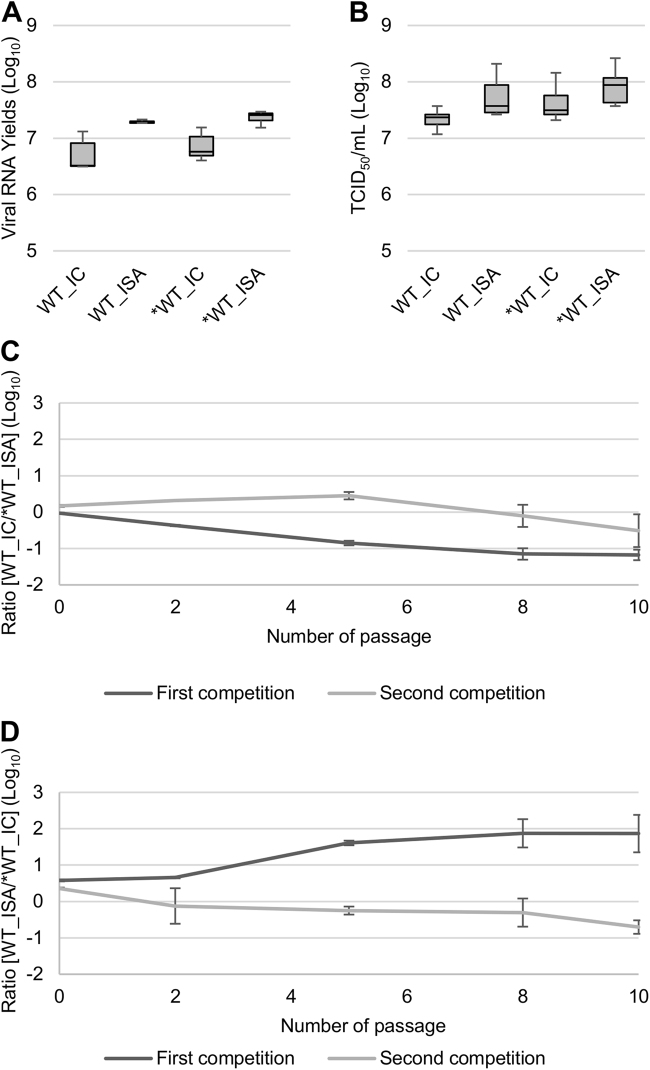
Impact of the ISA method on replicative fitness. Viral loads in cell supernatant media, after one passage in Vero ATCC cells, were estimated using a real-time RT-PCR assay (molecular viral loads, **a** and a TCID_50_ assay (infectious titers), **b**. We then performed competition experiments **c**, **d**: WT and *WT viruses were mixed together to infect Vero ATCC cells. Cell supernatant media were passaged 10 times. The relative proportion of each virus was evaluated by sequencing a genomic fragment encompassing the differentiating position 4420. In **a**, **b**, the bottom and top of the box represent the first and third quartiles, the band inside the box represents the median value and error bars represent the minimum and maximum values. In **c**, **d**, the error bars represent the standard deviation

To evaluate the replicative fitness of the WT and *WT viruses, viral RNA and infectious viral loads were estimated in triplicate using qRT-PCR and TCID_50_, respectively. No significant differences in replicative fitness, according to the method used for generating the viruses, were detected using the Student and Wilcoxon tests (Fig. 2a, b).

Accordingly, a more sensitive method for detecting relative differences in replicative fitness between viruses was used. This method involved infecting Vero ATCC cells with a mixture at an initial TCID_50_ ratio of 50/50 of infectious WT and *WT viruses. In total, four competition tests were carried out (see details in the “Materials and Methods” section), each performed in triplicate. Recovered cell supernatant media were serially subcultured 10 times to enable the long-term follow-up of each viral population. The relative proportion of each virus was evaluated at passages 0, 2, 5, 8 and 10 by amplifying and sequencing a genomic fragment encompassing position 4420, which differentiates the viruses (results were expressed as log_10_ ratio of ISA virus/IC virus). Overall, no clear trend emerged (Fig. 2c, d): 3 competitions showed a slight advantage for IC viruses (mean final log_10_ ratio between −0.51 and −1.18), whereas ISA viruses predominated in one competition (mean final log_10_ ratio = 1.87). Importantly, we observed low variability within triplicates (represented as standard deviation in Fig. 2c, d).

#### Evolution of a low replicative fitness virus

The aforementioned experiments were performed with viruses displaying a wild-type phenotype and a high replicative fitness profile. Thus, these viruses were well adapted to cell culture conditions. In the next series of experiments, we used a mutated virus with lower replicative fitness in cellulo. This CHIKV strain (designated ΔWT virus) was previously produced by random genomic re-encoding, and its genome includes 882 synonymous mutations located in three coding regions^21^. It was formerly observed that the replicative fitness of the ΔWT virus increased during serial passage in Vero cells^21^.

**Fig. 3:**
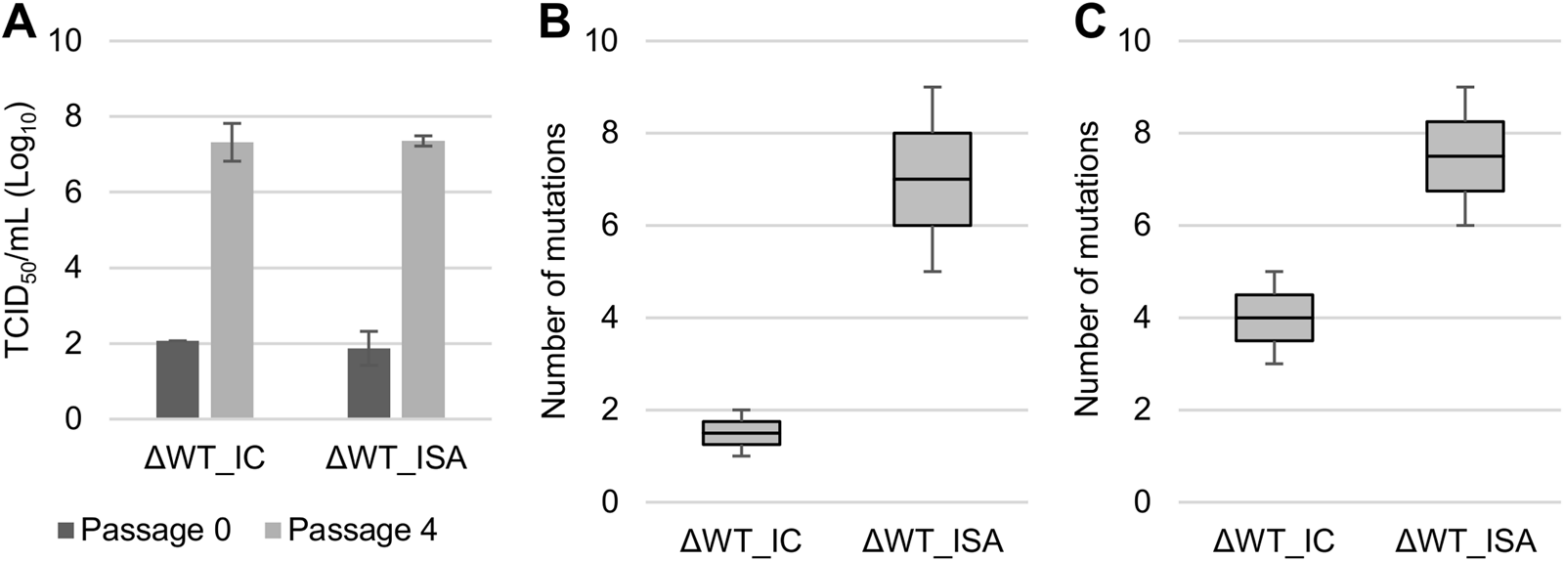
Impact of the ISA method on evolution of a low replicative fitness virus (ΔWT virus). Evolution of cell supernatant infectious virus titers (TCID_50_ assay) during passage in Vero ATCC cells (**a)**. **b**, **c** represent the number of mutations detected at the first and fourth passage, respectively. In **a**, the average number of TCID_50_/ml is shown, and error bars represent the standard deviation. In **b**, **c**, the bottom and top of the box represent the first and third quartiles, the band inside the box represents the median value, and the error bars represent the minimum and maximum values

We investigated the impact of the use of the ISA method in developing this strain on its evolution during serial passage in cell culture. We observed that the infectious titers increased with passage, and the rate of increase was similar for the ΔWT_IC and ΔWT_ISA viruses (Fig. 3a). Genome sequences (established in duplicate at the first and fourth passages) revealed a higher mutation rate in the ΔWT_ISA viruses than in the ΔWT_IC viruses (Fig. 3b, c). For each replicate of the ΔWT_IC virus, at the fourth passage, one fixed mutation was identified (5467 G > A and 1597 C > T, respectively, for replicate #1 (frequency: 87.2%) and #2 (frequency: 99.7%)), and a progressive increase of low-frequency mutations between the first and fourth passages was observed. The evolutionary patterns were different for the ΔWT_ISA viruses: mutation frequencies fluctuated over time but always remained under 73% (Supplementary Figure S4).

### Generating viruses using a new ISA-derived method: SuPReMe

On the basis of the above-described results, we attempted to develop an ISA-derived method that generated clonal populations of viruses. Therefore, the two main objectives were as follows: (i) to decrease the genetic diversity of viruses produced by the ISA method and (ii) to conserve the striking advantages of the ISA method, which include the use of subgenomic DNA fragments and the rapidity, flexibility and versatility of the technique.

#### Implementation

**Fig. 4:**
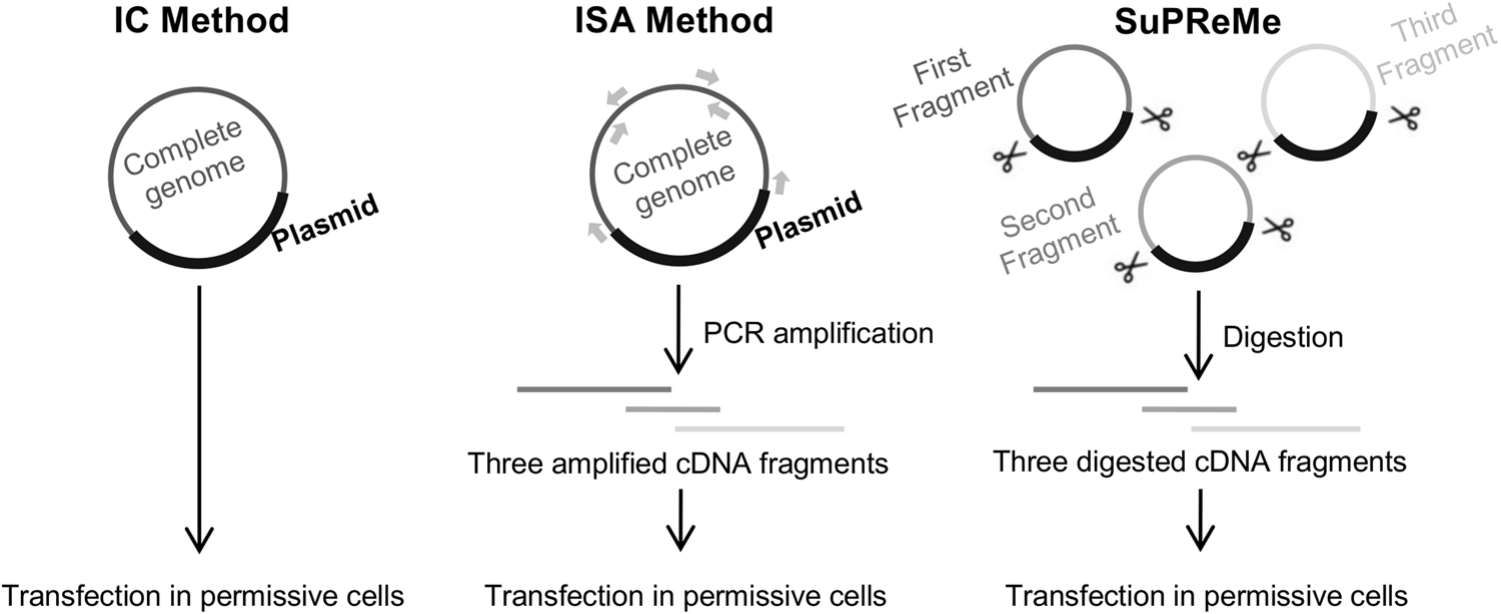
General overview of reverse genetics methods presented in this study. The SuPReMe comprises the following steps: -Cloning subgenomic overlapping DNA fragments flanked by two unique restriction sites. -Digesting each cloned subgenomic overlapping DNA fragments by restriction enzymes. -Preparing an equimolar mix of digested subgenomic overlapping DNA fragments. -Using that mix for cell transfection

The main difference between SuPReMe and the original ISA method is determined by the nature of the DNA fragments used for cell transfection. To ensure the clonality of these subgenomic fragments, SuPReMe uses digested plasmids rather than PCR amplicons. Prior to digestion, each subgenomic overlapping DNA fragment was cloned into a bacterial plasmid with unique restriction sites added at the 5′ and 3′ ends of the subgenomic region. The steps for preparing an equimolar mix and performing cell transfection were identical to those steps in the ISA procedure (Fig. 4).

We tested this procedure with the *WT virus. The DNA amplicons used for the ISA method were cloned into bacterial plasmids with NotI and AvrII restriction sites added at the 5′ and 3′ ends of each DNA fragment, respectively. Then, we applied the SuPReMe as described above. The transfection and virology analyses were similar to those described in previous sections. The viruses produced using the SuPReMe were designated *WT_SPR viruses. Two independent experiments, each consisting of 3 transfections, were performed.

#### Evaluation of genetic diversity

To evaluate the impact of the SuPReMe on the genetic diversity of viral populations, the genome sequences of the *WT_SPR viruses (established in triplicate using next-generation sequencing) were compared with those of the *WT_IC and *WT_ISA viruses.

**Fig. 5:**
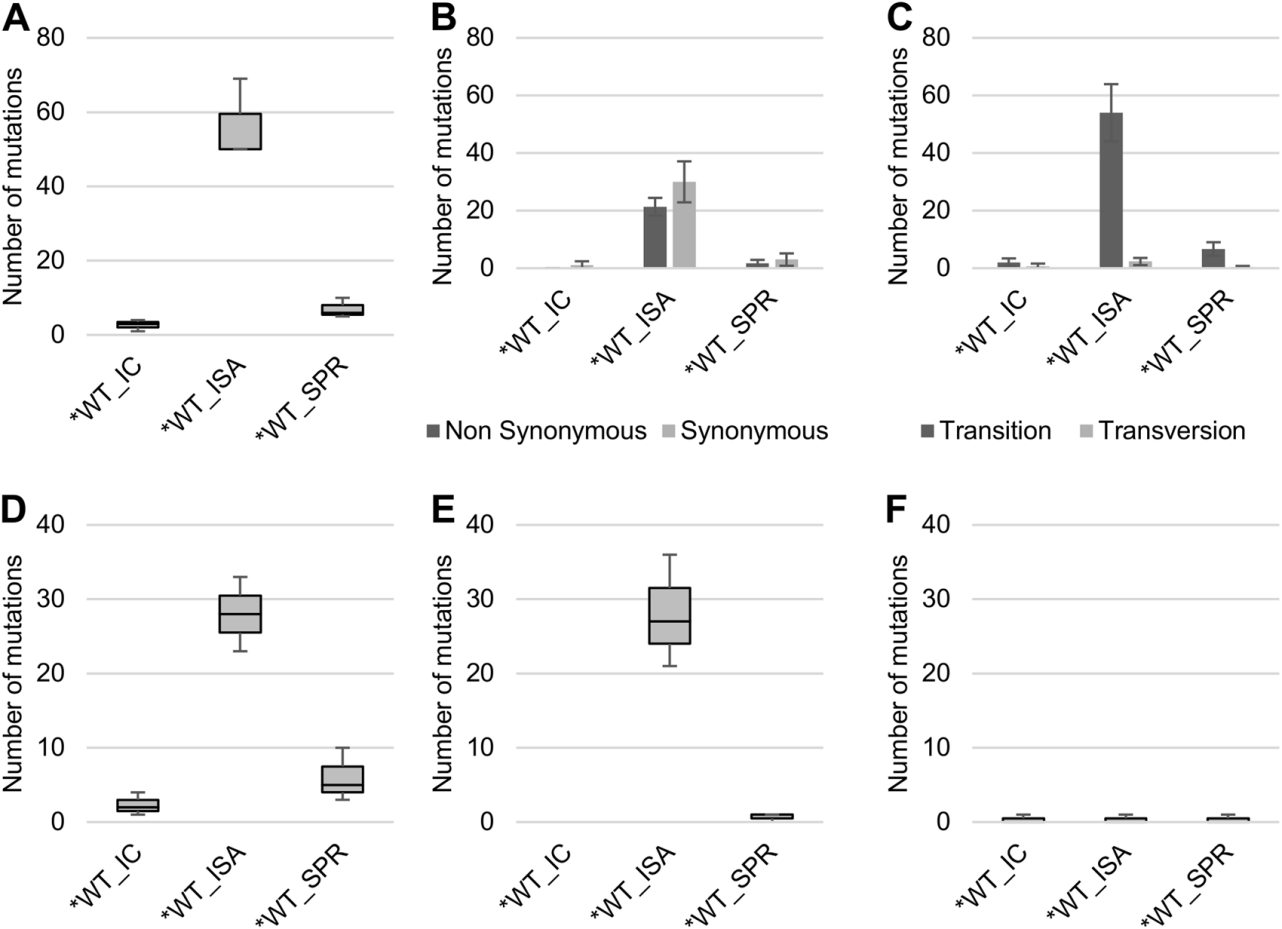
Impact of SuPReMe on genetic diversity of viral populations. To investigate the impact of the reverse genetics method on the genetic heterogeneity of the viral populations, the complete genome sequence of each virus was established in triplicate. **a** represents the number of mutations detected. Mutation characteristics are represented in **b** (nonsynonymous/synonymous mutations) and **c** (transition/transversion). **d**–**f** represent the number of low-, mid- and high-frequency mutations, respectively. In **a**, **d**–**f**, the bottom and top of the box are the first and third quartiles, the band inside the box represents the median value and error bars represent the minimum and maximum values. In **b**, **c**, the average number of mutations is shown, and error bars represent the standard deviation

Viruses generated using the SuPReMe exhibited a lower average number of mutations than those produced using the ISA method (7 vs. 57.33; Student's test: *p* = 0.0016; Fig. 5a). This average number was higher than that obtained with viruses produced using ICs, but the difference was insignificant (7 vs. 2.67; Student's test: *p* = 0.07; Fig. 5a). The mutation characteristics (*i*.e., synonymous/nonsynonymous and transition/transversion) were similar, irrespective of the reverse genetics method used to generate the viruses (Fig. 5b, c). Mutations in the*WT_SPR viruses were distributed throughout the complete genome, but, in a manner reminiscent of what had been previously observed for IC-derived viruses, a high proportion of these mutations was located in the 5′-UTR (5.3%; 3/19; Supplementary Figure S5). Moreover, the average numbers of low and mid-frequency mutations were lower than those of the *WT_ISA viruses and slightly higher than those of the *WT_IC viruses (Fig. 5d, e). The average number of high-frequency mutations was similar for all viruses. The proportion of mutations shared between triplicates of the same virus ranged between those of the *WT_IC and *WT_ISA viruses (Supplementary Figure S6).

#### Evaluation of replicative fitness in cellulo

**Fig. 6:**
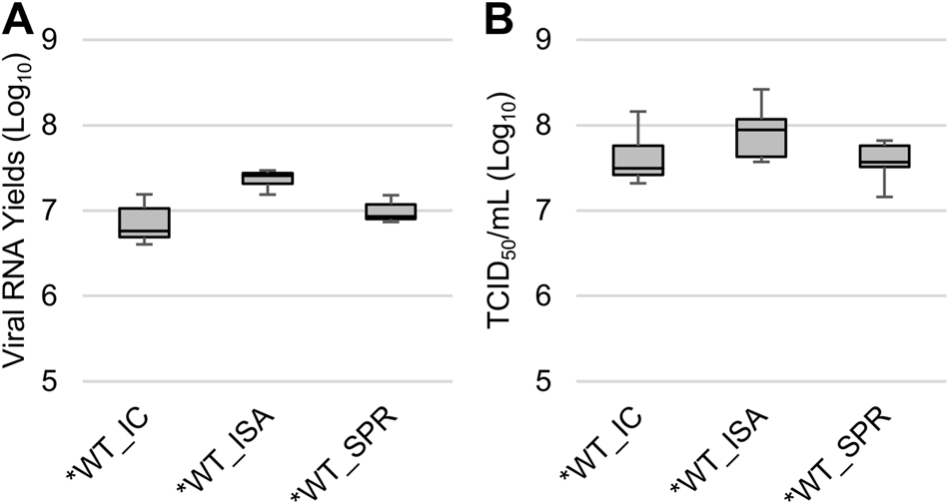
Impact of the SuPReMe on replicative fitness. Cell supernatant media after one passage on Vero ATCC cells were used to measure viral loads using a real-time RT-PCR assay (molecular viral loads, **a**) and a TCID_50_ assay (infectious titers, **b**). The bottom and top of the box represent the first and third quartiles, the band inside the box represents the median value, and error bars represent the minimum and maximum values

When the relative replicative fitness of the viruses derived by the three methods was compared in cellulo, there were no significant differences in the molecular and infectious viral loads (Student and Wilcoxon tests; Fig. 6).

## Discussion

The use of plasmids containing specified full-length genomes has revolutionized the study of positive-sensesingle-stranded RNA viruses^2^. Once such clones are obtained, they represent a convenient tool to study the viruses, and, in particular, they allow for finely controlled mutagenesis studies, which can associate the biological properties of the viruses with specific proteins or genetic traits. However, full-length genomes cannot always be stably maintained in plasmids^1^. Thus, in such cases, these plasmids may require modification to increase their stability^25^.

Bacterium-free techniques do not have this limitation^9–11^. For example, the ISA method is a technological breakthrough for the production of recombinant positive-sense single-stranded RNA viruses, since the reconstruction of full-length genomes occurs in cellulo following the transfection of subgenomic amplicons^12^.

Consequently, the major objective of this study was to develop an ISA-based procedure to generate clonal populations of recombinant viruses. We hypothesized that the original ISA protocol would not be suitable to achieve this goal because that protocol is associated with PCR-induced viral heterogeneity. Indeed, during the PCR amplification of subgenomic amplicons, thermostable DNA-dependent DNA polymerases induce levels of molecular heterogeneity that vary according to their relative copying “fidelity” (i.e., their specific rate of error-prone nucleotide incorporation)^17^.To confirm this assumption, we first explored the genotypic and phenotypic characteristics of CHIKV generated using either the ISA- or IC-based reverse genetics methods^12^.

Not surprisingly, the transfection of CHIKV infectious clones into permissive cells led to the production of a genetically “clonal” viral population. In contrast, the use of PCR products, even though they were produced using “high-fidelity” polymerases, generated higher genetic diversity within the viral population.

Since these studies require the replication of viruses in cell culture, the genomic variation linked with the molecular stages of the reverse genetics protocol cannot be totally dissociated from early evolutionary events (i.e., stochastic mutations during viral replication and/or selection of adaptive mutations). Therefore, the genetic variability observed with viruses rescued using ICs was likely the consequence of early adaptation to culture conditions, since specific mutational hot spots and higher proportions of shared mutations were observed, as previously described^26^.

Our results show that during early passages in cell culture, the variability observed in almost all viruses produced from PCR amplicons was randomly distributed throughout the genome and may reflect, in part, the heterogeneity of the molecular populations used for transfection.

In some cases, such genomic variability may facilitate the adaptation of RNA viruses^27,28^. Therefore, we investigated whether the observed genotypic differences between populations of ISA- and IC-derived viruses could be associated with variation in replicative fitness in vitro. Initially, the amounts of viral RNA and infectious titers in the supernatant media were found to be equivalent. Then, using a more sensitive method, namely, competition experiments, we detected minor fluctuations in fitness, most likely in relation to slight variations in the experimental conditions. Furthermore, the ISA method was not found to impact the evolution of a re-encoded CHIKV strain. We concluded that the differences between the genomic sequences of ISA- or IC-derived virus populations were associated with no or insignificant phenotypic differences in vitro, validating the possible use of both methods for in vitro analyses. However, it has been shown previously that slight differences in replicative patterns in vitro could be associated with significant differences in replicative fitness in vivo^29,30^. Therefore, our results should be interpreted cautiously and do not imply that in vivo phenotypes would be identical for ISA- and IC-derived viruses.

Accordingly, we developed a reverse genetics technique that combined the simplicity of ISA with its applicability and reproducibility for a large range of viruses and its ability to produce the quasi-clonality of infectious clone-derived protocols. By replacing the overlapping subgenomic amplicons used in the ISA method with corresponding DNA fragments derived from the digestion of plasmids containing the same subgenomic sequences, we were able to generate infectious viruses. Moreover, the genetic diversity of the viral populations derived using this new SuPReMe protocol was very low and comparable with that of viruses rescued using infectious clones, rather than that of viruses issued from the ISA method. Nevertheless, the genetic heterogeneity associated with the SuPReMe protocol remained slightly higher than that found in IC-derived viruses; the mechanisms underlying this difference remains undetermined, since both methods rely on the use of plasmids.

The ISA method allows for the production of infectious viruses within days, whereas the time required for the construction of an infectious clone is highly variable and unpredictable (usually several months)^1,12^. Since biotechnology companies are now able to de novo synthesize non-infectious plasmids containing sub-genomic overlapping fragments in 4–6 weeks, SuPReMe may enable the generation of clonal populations of infectious viruses within 1–2 months. This strategy is also more suitable for subsequent mutagenesis experiments, since the modification of one fragment is generally needed to introduce point mutations.

In conclusion, we demonstrated that PCR amplification of subgenomic DNA fragments during the ISA procedure triggered artificial genotypic diversity of the rescued viral populations. We, nevertheless,demonstrated that viruses generated using the ISA method or infectious clones exhibited similar replicative fitness when compared in vitro. SuPReMe, a new ISA-derived method using subgenomic plasmid sequences instead of amplicons, was shown to retain the major advantages of the original ISA method, such as its rapidity, flexibility and versatility, while producing quasi-clonal populations of viruses as infectious clones. The efficacy of this method should be demonstrated with other viruses, such as flaviviruses, which are known to be difficult to manipulate using reverse genetics methods^1^.

In conclusion, this method appears to represent a highly attractive alternative to the direct use of infectious clones for obtaining virus populations with relatively limited genomic heterogeneity.

## Electronic supplementary material

Supplementary Figure S6(TIF 93 kb)

Supplementary Table S1(PDF 537 kb)

Supplementary Figure S1(TIF 295 kb)

Supplementary Figure S2(TIF 620 kb)

Supplementary Figure S3(TIF 96 kb)

Supplementary Figure S4(TIF 369 kb)

Supplementary Figure S5(TIF 244 kb)
