# Supplementary figures and images for: SuPReMe: a rapid reverse genetics method to generate clonal populations of recombinant RNA viruses
Source: Emerg Microbes Infect. 2018 Mar 21;7:40. doi: 10.1038/s41426-018-0040-2 (PMC5861084; doi:10.1038/s41426-018-0040-2)

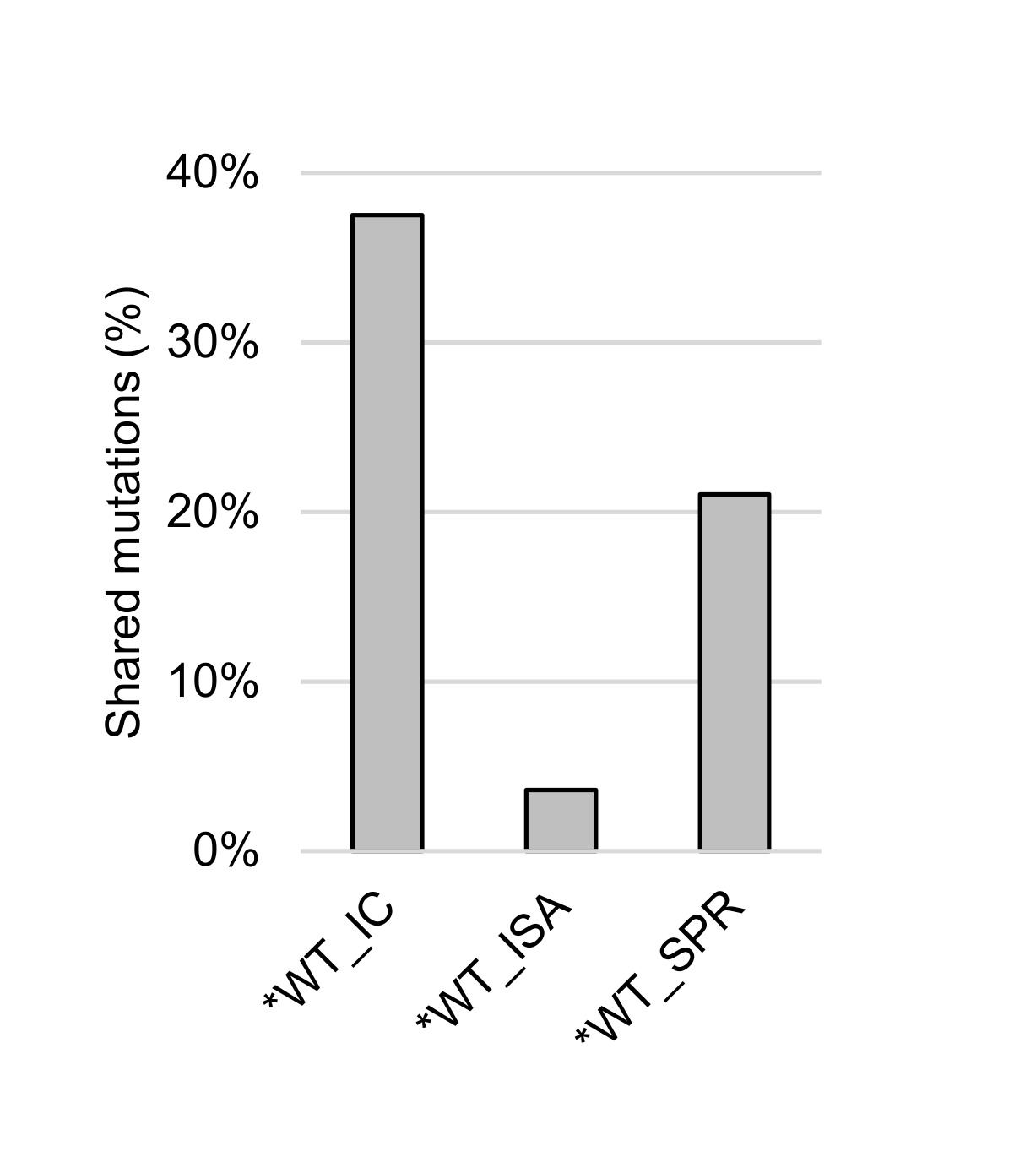

Supplement: Supplementary file 1 — Supplementary Figure S6(TIF 93 kb) [file 41426_2018_40_MOESM1_ESM.tif]

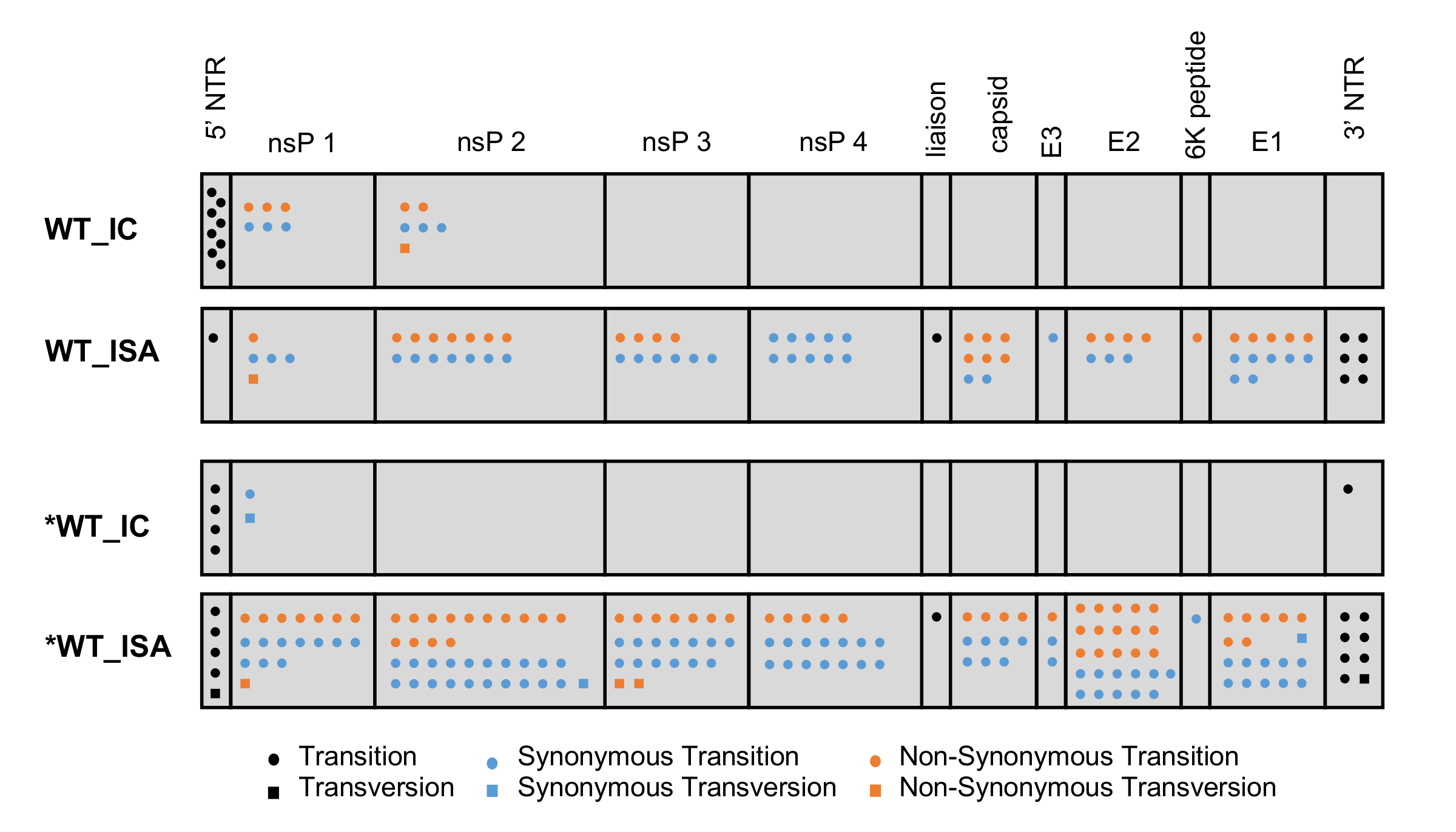

Supplement: Supplementary file 3 — Supplementary Figure S1(TIF 295 kb) [file 41426_2018_40_MOESM3_ESM.tif]

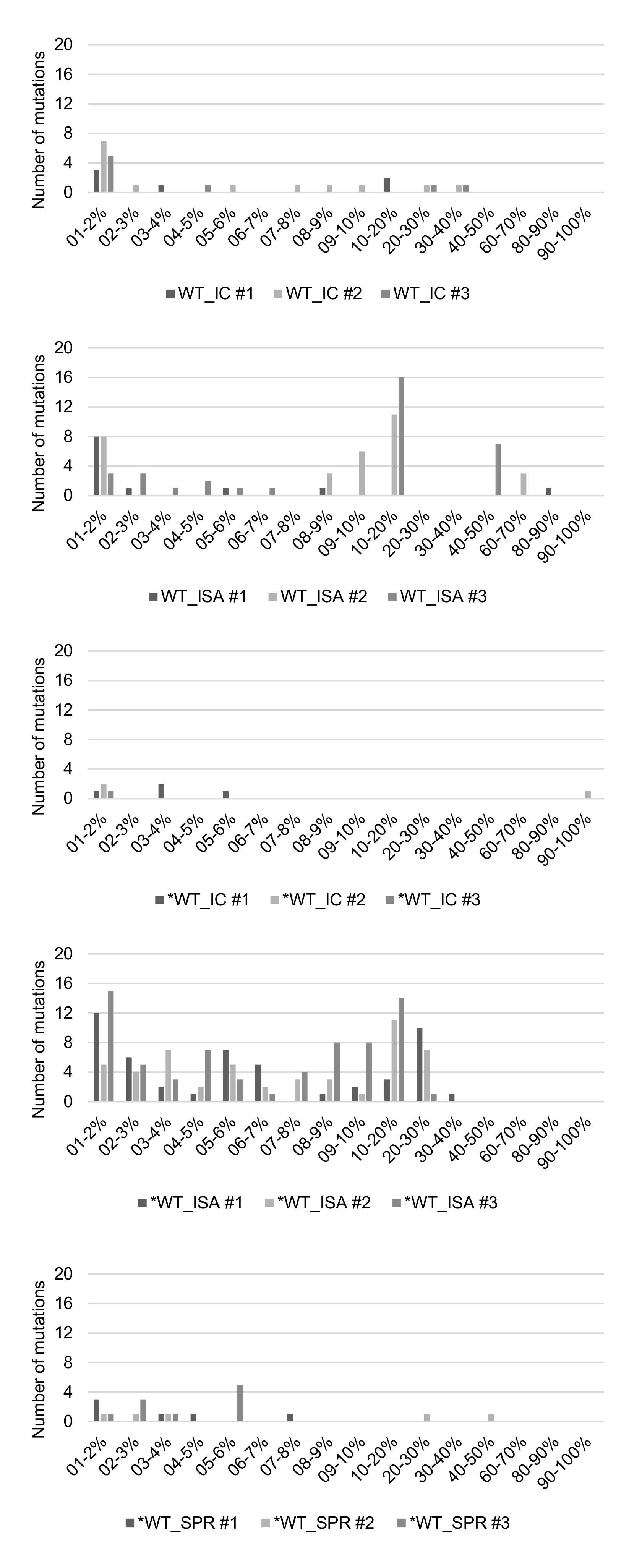

Supplement: Supplementary file 4 — Supplementary Figure S2(TIF 620 kb) [file 41426_2018_40_MOESM4_ESM.tif]

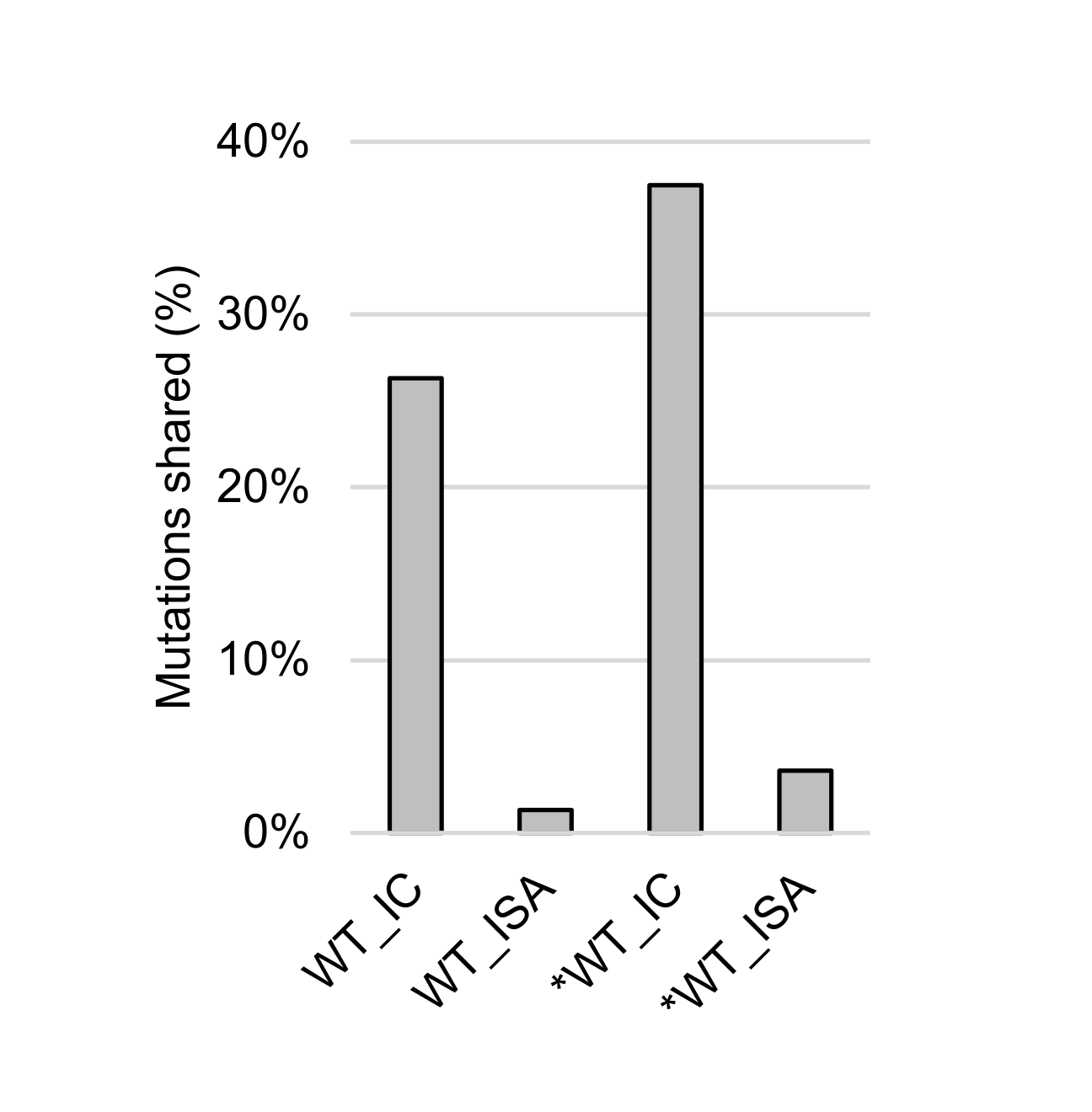

Supplement: Supplementary file 5 — Supplementary Figure S3(TIF 96 kb) [file 41426_2018_40_MOESM5_ESM.tif]

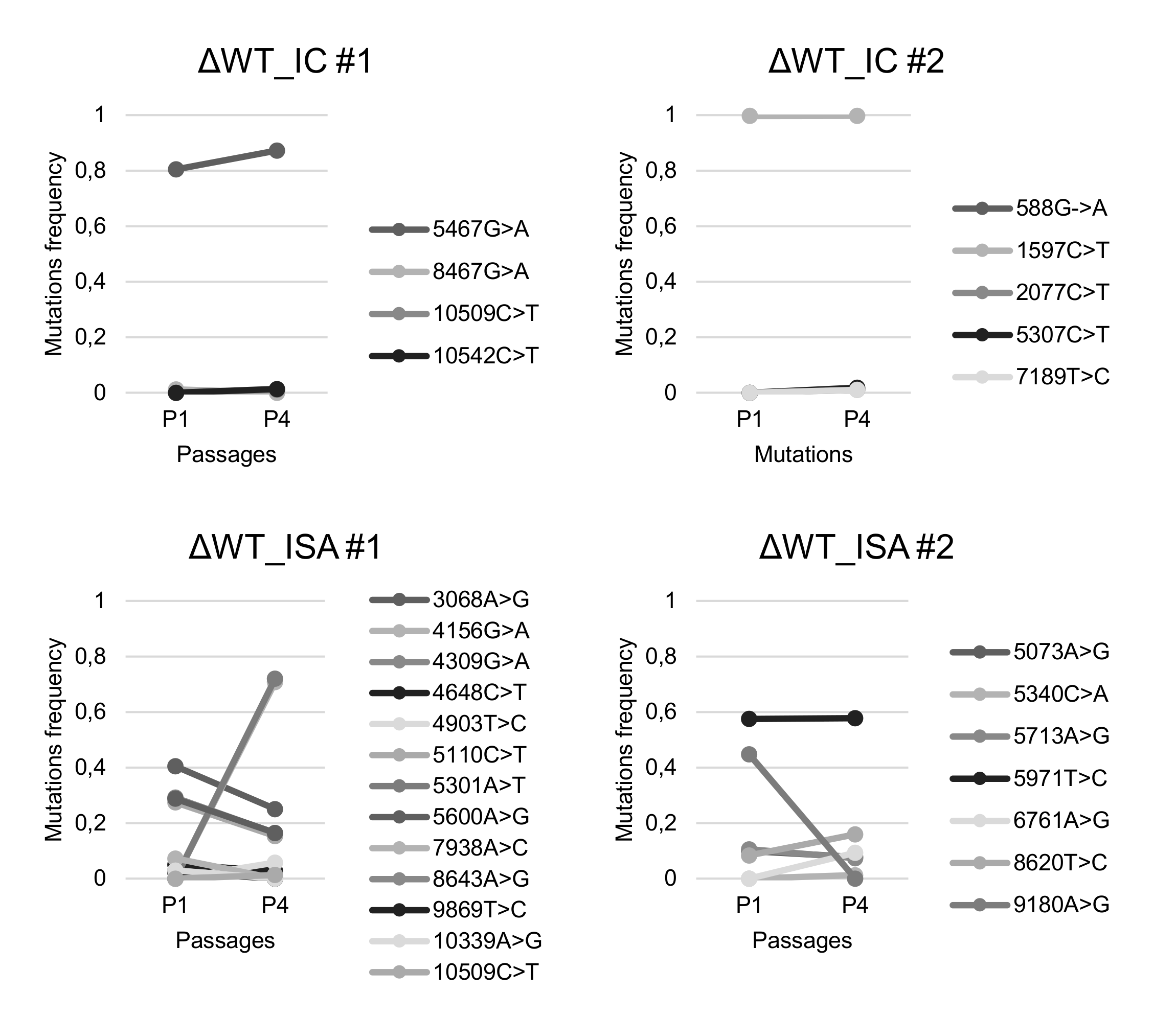

Supplement: Supplementary file 6 — Supplementary Figure S4(TIF 369 kb) [file 41426_2018_40_MOESM6_ESM.tif]

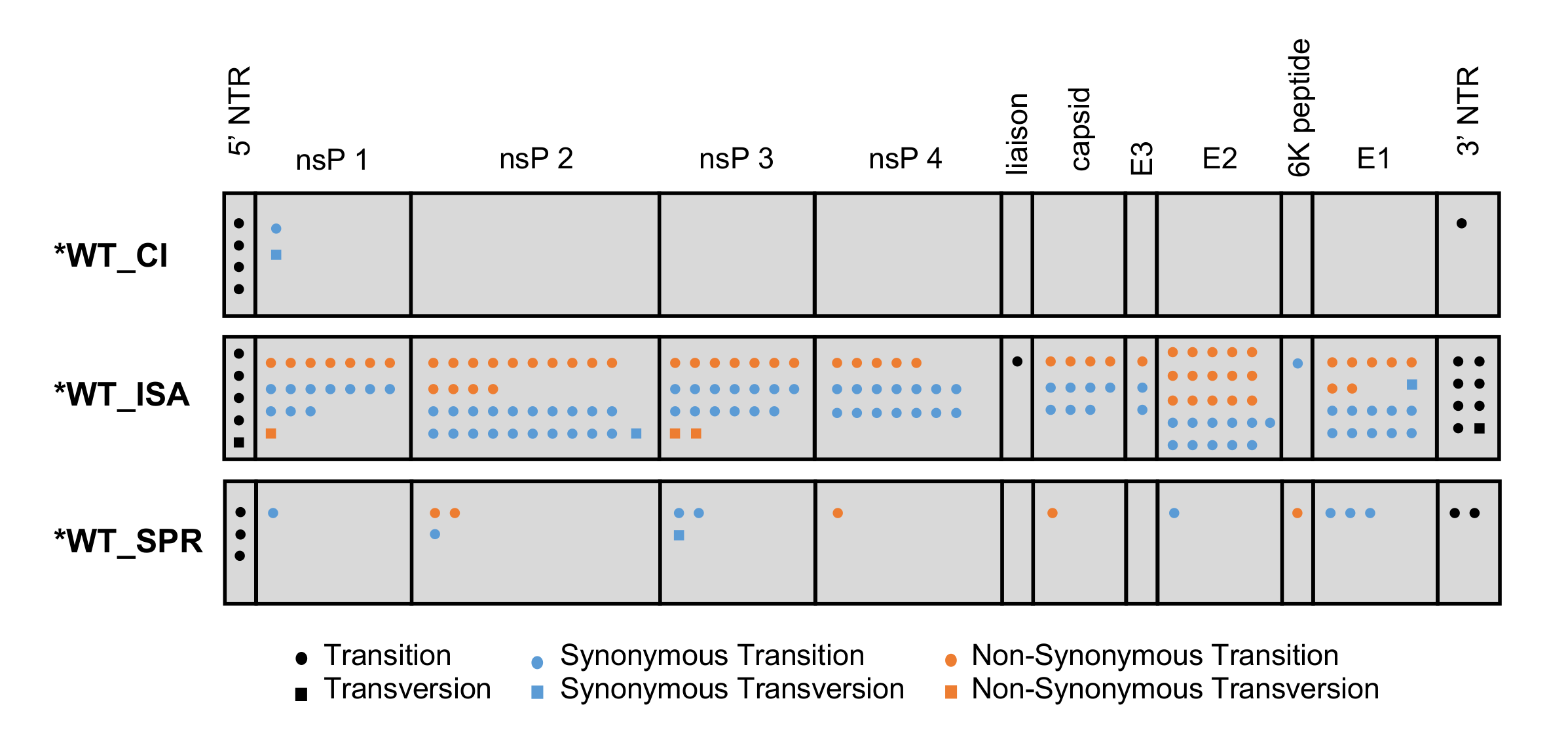

Supplement: Supplementary file 7 — Supplementary Figure S5(TIF 244 kb) [file 41426_2018_40_MOESM7_ESM.tif]
