# Supplementary material for: SuPReMe: a rapid reverse genetics method to generate clonal populations of recombinant RNA viruses
Source: Emerg Microbes Infect. 2018 Mar 21;7:40. doi: 10.1038/s41426-018-0040-2 (PMC5861084; doi:10.1038/s41426-018-0040-2)
Supplement: Supplementary file 2 — Supplementary Table S1(PDF 537 kb) [file 41426_2018_40_MOESM2_ESM.pdf]

Virus	Mutation position	Region	Frequency	Nucleotide	Transition / Transversion	Amino acid	Synonymous / Non-Synonymous
*WT_IC #1	23	5'NTR	0,037	G>A	Transition		
*WT_IC #1	20	5'NTR	0,016	G>A	Transition		
*WT_IC #1	24	5'NTR	0,035	C>T	Transition		
*WT_IC #1	22	5'NTR	0,052	A>G	Transition		
*WT_IC #2	283	nsP1	0,013	A>C	Transversion	Ala	Synonymous
*WT_IC #2	1561	nsP1	0,011	A>G	Transition	Lys	Synonymous
*WT_IC #2	4420	nsP3	0,968	C>A	Transversion	Ser	Synonymous
*WT_IC #3	11761	3'NTR	0,015	T>C	Transition		
*WT_ISA #1	22	5'NTR	0,013	A>G	Transition		
*WT_ISA #1	1312	nsP1	0,243	A>G	Transition	Glu	Synonymous
*WT_ISA #1	496	nsP1	0,264	A>G	Transition	Ser	Synonymous
*WT_ISA #1	1323	nsP1	0,090	C>T	Transition	Thr > Ile	Non-Synonymous
*WT_ISA #1	1400	nsP1	0,013	A>G	Transition	Lys > Glu	Non-Synonymous
*WT_ISA #1	1909	nsP2	0,157	A>G	Transition	Ala	Synonymous
*WT_ISA #1	2131	nsP2	0,250	G>A	Transition	Leu	Synonymous
*WT_ISA #1	2345	nsP2	0,306	A>G	Transition	Met > Val	Non-Synonymous
*WT_ISA #1	3016	nsP2	0,066	A>G	Transition	Ala	Synonymous
*WT_ISA #1	3131	nsP2	0,012	G>A	Transition	Val > Ile	Non-Synonymous
*WT_ISA #1	3291	nsP2	0,249	A>G	Transition	Lys > Arg	Non-Synonymous
*WT_ISA #1	3643	nsP2	0,023	A>G	Transition	Val	Synonymous
*WT_ISA #1	3664	nsP2	0,066	A>G	Transition	Gly	Synonymous
*WT_ISA #1	3831	nsP2	0,091	A>G	Transition	Lys > Arg	Non-Synonymous
*WT_ISA #1	4120	nsP3	0,024	T>G	Transversion	Asp > Glu	Non-Synonymous
*WT_ISA #1	4192	nsP3	0,056	A>C	Transversion	Lys > Asn	Non-Synonymous
*WT_ISA #1	4903	nsP3	0,269	T>C	Transition	Leu	Synonymous
*WT_ISA #1	5083	nsP3	0,167	A>G	Transition	Thr	Synonymous
*WT_ISA #1	5147	nsP3	0,054	T>C	Transition	Ser > Pro	Non-Synonymous
*WT_ISA #1	5170	nsP3	0,054	A>G	Transition	Pro	Synonymous
*WT_ISA #1	5249	nsP3	0,165	T>C	Transition	Trp > Arg	Non-Synonymous
*WT_ISA #1	5396	nsP3	0,057	A>G	Transition	Thr > Ala	Non-Synonymous
*WT_ISA #1	5630	nsP3	0,242	A>G	Transition	Thr > Ala	Non-Synonymous
*WT_ISA #1	5917	nsP4	0,030	T>C	Transition	Cys	Synonymous
*WT_ISA #1	5944	nsP4	0,058	A>G	Transition	Pro	Synonymous
*WT_ISA #1	5982	nsP4	0,015	T>C	Transition	Val > Ala	Non-Synonymous
*WT_ISA #1	6252	nsP4	0,049	A>G	Transition	Gln > Arg	Non-Synonymous
*WT_ISA #1	6457	nsP4	0,011	A>G	Transition	Lys	Synonymous
*WT_ISA #1	6827	nsP4	0,055	T>C	Transition	Leu	Synonymous
*WT_ISA #1	7720	capsid	0,062	A>G	Transition	Thr > Ala	Non-Synonymous
*WT_ISA #1	7849	capsid	0,020	A>G	Transition	Lys > Glu	Non-Synonymous
*WT_ISA #1	7896	capsid	0,060	A>G	Transition	Glu	Synonymous
*WT_ISA #1	8476	E3	0,290	A>G	Transition	Met > Val	Non-Synonymous
*WT_ISA #1	8520	E3	0,014	T>C	Transition	Cys	Synonymous
*WT_ISA #1	8779	E2	0,027	A>G	Transition	Arg > Gly	Non-Synonymous
*WT_ISA #1	8862	E2	0,069	A>G	Transition	Lys	Synonymous
*WT_ISA #1	8891	E2	0,270	A>G	Transition	Asp > Gly	Non-Synonymous
*WT_ISA #1	9180	E2	0,013	A>G	Transition	Thr	Synonymous
*WT_ISA #1	9235	E2	0,012	C>T	Transition	His > Tyr	Non-Synonymous
*WT_ISA #1	9459	E2	0,010	T>C	Transition	Tyr	Synonymous
*WT_ISA #1	9714	E2	0,010	T>C	Transition	Cys	Synonymous

*WT_ISA #1	9799	E2	0,063	A>G	Transition	Thr > Ala	Non-Synonymous
*WT_ISA #1	10154	E1	0,024	T>C	Transition	Val > Ala	Non-Synonymous
*WT_ISA #1	10383	E1	0,263	A>G	Transition	Ser	Synonymous
*WT_ISA #1	10506	E1	0,033	A>G	Transition	Thr	Synonymous
*WT_ISA #1	11033	E1	0,260	A>G	Transition	Glu > Gly	Non-Synonymous
*WT_ISA #1	11073	E1	0,094	A>G	Transition	Leu	Synonymous
*WT_ISA #1	11151	E1	0,014	C>T	Transition	His	Synonymous
*WT_ISA #1	11273	E1	0,012	T>C	Transition	Ile > Thr	Non-Synonymous
*WT_ISA #1	11473	3'NTR	0,029	T>C	Transition		
*WT_ISA #2	399	nsP1	0,231	T>C	Transition	Leu > Pro	Non-Synonymous
*WT_ISA #2	811	nsP1	0,026	A>G	Transition	Ser	Synonymous
*WT_ISA #2	69	5'NTR	0,127	A>G	Transition		
*WT_ISA #2	1237	nsP1	0,037	C>T	Transition	Val	Synonymous
*WT_ISA #2	1347	nsP1	0,152	A>T	Transversion	Lys > Met	Non-Synonymous
*WT_ISA #2	1551	nsP1	0,021	A>G	Transition	Asp > Gly	Non-Synonymous
*WT_ISA #2	1988	nsP2	0,162	C>T	Transition	Leu	Synonymous
*WT_ISA #2	2332	nsP2	0,129	C>T	Transition	Ile	Synonymous
*WT_ISA #2	2526	nsP2	0,162	A>G	Transition	Lys > Arg	Non-Synonymous
*WT_ISA #2	2995	nsP2	0,226	A>G	Transition	Pro	Synonymous
*WT_ISA #2	3160	nsP2	0,096	A>G	Transition	Lys	Synonymous
*WT_ISA #2	3205	nsP2	0,130	A>G	Transition	Glu	Synonymous
*WT_ISA #2	3321	nsP2	0,010	A>G	Transition	Asn > Ser	Non-Synonymous
*WT_ISA #2	3376	nsP2	0,039	A>G	Transition	Ala	Synonymous
*WT_ISA #2	3556	nsP2	0,055	A>G	Transition	Glu	Synonymous
*WT_ISA #2	3678	nsP2	0,018	A>G	Transition	Tyr > Cys	Non-Synonymous
*WT_ISA #2	3928	nsP2	0,033	A>G	Transition	Arg	Synonymous
*WT_ISA #2	3936	nsP2	0,029	A>G	Transition	Lys > Arg	Non-Synonymous
*WT_ISA #2	3943	nsP2	0,054	A>T	Transversion	Pro	Synonymous
*WT_ISA #2	4290	nsP3	0,079	A>G	Transition	Lys	Synonymous
*WT_ISA #2	4411	nsP3	0,013	T>C	Transition	Gly	Synonymous
*WT_ISA #2	4891	nsP3	0,082	C>T	Transition	Arg	Synonymous
*WT_ISA #2	5038	nsP3	0,090	A>G	Transition	Pro	Synonymous
*WT_ISA #2	5396	nsP3	0,123	A>G	Transition	Thr > Ala	Non-Synonymous
*WT_ISA #2	5604	nsP3	0,224	A>G	Transition	Asp > Gly	Non-Synonymous
*WT_ISA #2	5739	nsP4	0,063	A>G	Transition	Val	Synonymous
*WT_ISA #2	5818	nsP4	0,124	A>G	Transition	Lys	Synonymous
*WT_ISA #2	6196	nsP4	0,076	T>C	Transition	Ala	Synonymous
*WT_ISA #2	6761	nsP4	0,060	A>G	Transition	Thr > Ala	Non-Synonymous
*WT_ISA #2	6857	nsP4	0,018	C>T	Transition	His > Tyr	Non-Synonymous
*WT_ISA #2	7222	nsP4	0,235	T>C	Transition	Ala	Synonymous
*WT_ISA #2	7503	liaison	0,121	T>C	Transition		
*WT_ISA #2	7905	capsid	0,035	T>C	Transition	Cys	Synonymous
*WT_ISA #2	7974	capsid	0,040	A>G	Transition	Lys	Synonymous
*WT_ISA #2	8321	capsid	0,079	A>G	Transition	Lys > Arg	Non-Synonymous
*WT_ISA #2	8625	E2	0,059	C>T	Transition	Cys	Synonymous
*WT_ISA #2	9164	E2	0,283	A>G	Transition	Glu > Gly	Non-Synonymous
*WT_ISA #2	9311	E2	0,051	T>C	Transition	Ile > Thr	Non-Synonymous
*WT_ISA #2	9334	E2	0,033	A>G	Transition	Thr > Ala	Non-Synonymous
*WT_ISA #2	9447	E2	0,027	A>G	Transition	Glu	Synonymous
*WT_ISA #2	10311	E1	0,279	A>C	Transversion	Ala	Synonymous
*WT_ISA #2	11041	E1	0,040	T>C	Transition	Ser > Pro	Non-Synonymous
*WT_ISA #2	11106	E1	0,089	T>C	Transition	Ser	Synonymous

*WT_ISA #2	11172	E1	0,033	A>G	Transition	Ser	Synonymous
*WT_ISA #2	11179	E1	0,033	A>G	Transition	Thr > Ala	Non-Synonymous
*WT_ISA #2	11316	3'NTR	0,289	T>C	Transition		
*WT_ISA #2	11320	3'NTR	0,063	A>G	Transition		
*WT_ISA #2	11601	3'NTR	0,122	A>G	Transition		
*WT_ISA #2	11703	3'NTR	0,111	C>A	Transversion		
*WT_ISA #2	11717	3'NTR	0,012	A>G	Transition		
*WT_ISA #3	25	5'NTR	0,023	C>T	Transition		
*WT_ISA #3	23	5'NTR	0,151	G>T	Transversion		
*WT_ISA #3	646	nsP1	0,024	T>C	Transition	Gly	Synonymous
*WT_ISA #3	747	nsP1	0,062	A>G	Transition	Lys > Arg	Non-Synonymous
*WT_ISA #3	1096	nsP1	0,095	T>C	Transition	Asp	Synonymous
*WT_ISA #3	1057	nsP1	0,010	A>G	Transition	Ser	Synonymous
*WT_ISA #3	22	5'NTR	0,149	A>G	Transition		
*WT_ISA #3	24	5'NTR	0,080	C>T	Transition		
*WT_ISA #3	190	nsP1	0,160	T>C	Transition	Ala	Synonymous
*WT_ISA #3	352	nsP1	0,081	A>G	Transition	Arg	Synonymous
*WT_ISA #3	1526	nsP1	0,025	A>G	Transition	Ser > Gly	Non-Synonymous
*WT_ISA #3	1621	nsP1	0,093	A>G	Transition	Ala	Synonymous
*WT_ISA #3	1650	nsP1	0,013	A>G	Transition	Asp > Gly	Non-Synonymous
*WT_ISA #3	1694	nsP2	0,102	A>G	Transition	Thr > Ala	Non-Synonymous
*WT_ISA #3	1737	nsP2	0,054	A>G	Transition	His > Arg	Non-Synonymous
*WT_ISA #3	1749	nsP2	0,074	A>G	Transition	Glu > Gly	Non-Synonymous
*WT_ISA #3	2359	nsP2	0,092	T>C	Transition	Gly	Synonymous
*WT_ISA #3	3397	nsP2	0,044	T>C	Transition	Tyr	Synonymous
*WT_ISA #3	3596	nsP2	0,185	G>A	Transition	Val > Ile	Non-Synonymous
*WT_ISA #3	3635	nsP2	0,086	A>G	Transition	Thr > Ala	Non-Synonymous
*WT_ISA #3	3738	nsP2	0,042	T>C	Transition	Ile > Thr	Non-Synonymous
*WT_ISA #3	3745	nsP2	0,123	A>G	Transition	Thr	Synonymous
*WT_ISA #3	3775	nsP2	0,140	C>T	Transition	Cys	Synonymous
*WT_ISA #3	3823	nsP2	0,095	A>G	Transition	Arg	Synonymous
*WT_ISA #3	3871	nsP2	0,123	A>G	Transition	Ala	Synonymous
*WT_ISA #3	3955	nsP2	0,081	C>T	Transition	Ser	Synonymous
*WT_ISA #3	4507	nsP3	0,102	C>T	Transition	Arg	Synonymous
*WT_ISA #3	4818	nsP3	0,043	T>C	Transition	Val > Ala	Non-Synonymous
*WT_ISA #3	5067	nsP3	0,084	C>T	Transition	Ala > Val	Non-Synonymous
*WT_ISA #3	5392	nsP3	0,013	A>G	Transition	Gln	Synonymous
*WT_ISA #3	5527	nsP3	0,124	A>G	Transition	Glu	Synonymous
*WT_ISA #3	5530	nsP3	0,011	A>G	Transition	Gly	Synonymous
*WT_ISA #3	5576	nsP3	0,012	T>C	Transition	Leu	Synonymous
*WT_ISA #3	5593	nsP3	0,012	T>C	Transition	Asp	Synonymous
*WT_ISA #3	5791	nsP4	0,035	A>G	Transition	Glu	Synonymous
*WT_ISA #3	6152	nsP4	0,016	A>G	Transition	Thr > Ala	Non-Synonymous
*WT_ISA #3	6250	nsP4	0,022	A>G	Transition	Leu	Synonymous
*WT_ISA #3	6481	nsP4	0,057	C>T	Transition	Thr	Synonymous
*WT_ISA #3	6862	nsP4	0,097	C>T	Transition	Ser	Synonymous
*WT_ISA #3	6940	nsP4	0,093	C>T	Transition	Gly	Synonymous
*WT_ISA #3	6985	nsP4	0,078	A>G	Transition	Thr	Synonymous
*WT_ISA #3	7647	capsid	0,135	C>T	Transition	Val	Synonymous
*WT_ISA #3	7745	capsid	0,012	A>G	Transition	Lys > Arg	Non-Synonymous
*WT_ISA #3	8232	capsid	0,137	A>G	Transition	Gly	Synonymous
*WT_ISA #3	8259	capsid	0,090	A>G	Transition	Gly	Synonymous

*WT_ISA #3	8343	capsid	0,037	A>G	Transition	Glu	Synonymous
*WT_ISA #3	8364	E3	0,015	A>G	Transition	Pro	Synonymous
*WT_ISA #3	8564	E2	0,087	T>C	Transition	Val > Ala	Non-Synonymous
*WT_ISA #3	8695	E2	0,084	T>C	Transition	Leu	Synonymous
*WT_ISA #3	8711	E2	0,013	A>G	Transition	Lys > Arg	Non-Synonymous
*WT_ISA #3	8779	E2	0,095	A>G	Transition	Arg > Gly	Non-Synonymous
*WT_ISA #3	8807	E2	0,012	C>T	Transition	Ala > Val	Non-Synonymous
*WT_ISA #3	8823	E2	0,078	T>C	Transition	Thr	Synonymous
*WT_ISA #3	8935	E2	0,130	G>A	Transition	Asp > Asn	Non-Synonymous
*WT_ISA #3	8988	E2	0,143	A>G	Transition	Lys	Synonymous
*WT_ISA #3	8997	E2	0,047	T>C	Transition	Pro	Synonymous
*WT_ISA #3	9232	E2	0,035	A>G	Transition	Asn > Asp	Non-Synonymous
*WT_ISA #3	9449	E2	0,012	A>G	Transition	Glu > Gly	Non-Synonymous
*WT_ISA #3	9450	E2	0,013	A>G	Transition	Glu	Synonymous
*WT_ISA #3	9593	E2	0,052	A>G	Transition	His > Arg	Non-Synonymous
*WT_ISA #3	9837	6k peptide	0,046	C>T	Transition	Tyr	Synonymous
*WT_ISA #3	10356	E1	0,012	A>G	Transition	Ala	Synonymous
*WT_ISA #3	10422	E1	0,027	T>C	Transition	Thr	Synonymous
*WT_ISA #3	10852	E1	0,048	T>C	Transition	Phe > Leu	Non-Synonymous
*WT_ISA #3	10917	E1	0,016	T>C	Transition	His	Synonymous
*WT_ISA #3	11034	E1	0,099	A>G	Transition	Glu	Synonymous
*WT_ISA #3	11080	E1	0,045	G>A	Transition	Ala > Thr	Non-Synonymous
*WT_ISA #3	11339	3'NTR	0,077	T>C	Transition		
*WT_ISA #3	11608	3'NTR	0,226	T>C	Transition		
*WT_SPR #1	24	5'NTR	0,032	C>T	Transition		
*WT_SPR #1	22	5'NTR	0,071	A>G	Transition		
*WT_SPR #1	1774	nsP2	0,013	C>T	Transition	Thr	Synonymous
*WT_SPR #1	4111	nsP3	0,011	G>C	Transversion	Ala	Synonymous
*WT_SPR #1	8898	E2	0,044	G>A	Transition	Arg	Synonymous
*WT_SPR #1	11277	E1	0,011	A>G	Transition	Leu	Synonymous
*WT_SPR #2	23	5'NTR	0,039	G>A	Transition		
*WT_SPR #2	24	5'NTR	0,222	C>T	Transition		
*WT_SPR #2	22	5'NTR	0,483	A>G	Transition		
*WT_SPR #2	2274	nsP2	0,010	A>G	Transition	Asn > Ser	Non-Synonymous
*WT_SPR #2	9935	6k peptide	0,026	G>A	Transition	Cys > Tyr	Non-Synonymous
*WT_SPR #3	1618	nsP1	0,038	G>A	Transition	Gln	Synonymous
*WT_SPR #3	3461	nsP2	0,027	G>A	Transition	Asp > Asn	Non-Synonymous
*WT_SPR #3	5587	nsP3	0,055	A>G	Transition	Glu	Synonymous
*WT_SPR #3	5626	nsP3	0,054	A>G	Transition	Ser	Synonymous
*WT_SPR #3	5709	nsP4	0,056	A>G	Transition	Lys > Arg	Non-Synonymous
*WT_SPR #3	7631	capsid	0,013	G>A	Transition	Arg > His	Non-Synonymous
*WT_SPR #3	11289	E1	0,027	A>G	Transition	Leu	Synonymous
*WT_SPR #3	11312	E1	0,024	A>G	Transition	STOP	Synonymous
*WT_SPR #3	11320	3'NTR	0,053	A>G	Transition		
*WT_SPR #3	11324	3'NTR	0,054	A>G	Transition		
WT_IC #1	22	5'NTR	0,150	A>G	Transition		
WT_IC #1	25	5'NTR	0,015	C>T	Transition		
WT_IC #1	545	nsP1	0,018	C>T	Transition	His > Tyr	Non-Synonymous
WT_IC #1	20	5'NTR	0,033	G>A	Transition		
WT_IC #1	23	5'NTR	0,012	G>A	Transition		
WT_IC #1	24	5'NTR	0,124	C>T	Transition		
WT_IC #2	24	5'NTR	0,279	C>T	Transition		

WT_IC #2	90	nsP1	0,013	A>G	Transition	Tyr > Cys	Non-Synonymous
WT_IC #2	883	nsP1	0,010	A>G	Transition	Lys	Synonymous
WT_IC #2	19	5'NTR	0,088	C>T	Transition		
WT_IC #2	20	5'NTR	0,093	G>A	Transition		
WT_IC #2	22	5'NTR	0,336	A>G	Transition		
WT_IC #2	23	5'NTR	0,016	G>A	Transition		
WT_IC #2	25	5'NTR	0,073	C>T	Transition		
WT_IC #2	1279	nsP1	0,058	C>T	Transition	Asp	Synonymous
WT_IC #2	2110	nsP2	0,021	G>T	Transversion	Lys > Asn	Non-Synonymous
WT_IC #2	2129	nsP2	0,011	C>T	Transition	Leu	Synonymous
WT_IC #2	2771	nsP2	0,013	G>A	Transition	Val > Ile	Non-Synonymous
WT_IC #2	2814	nsP2	0,011	C>T	Transition	Thr > Ile	Non-Synonymous
WT_IC #2	2917	nsP2	0,011	A>G	Transition	Val	Synonymous
WT_IC #3	764	nsP1	0,013	G>A	Transition	Gly > Arg	Non-Synonymous
WT_IC #3	22	5'NTR	0,228	A>G	Transition		
WT_IC #3	25	5'NTR	0,042	C>T	Transition		
WT_IC #3	1090	nsP1	0,011	T>C	Transition	Ile	Synonymous
WT_IC #3	24	5'NTR	0,349	C>T	Transition		
WT_IC #3	23	5'NTR	0,010	G>A	Transition		
WT_IC #3	55	5'NTR	0,011	A>G	Transition		
WT_IC #3	2834	nsP2	0,011	T>C	Transition	Leu	Synonymous
WT_ISA #1	1448	nsP1	0,016	T>A	Transversion	Ser > Thr	Non-Synonymous
WT_ISA #1	2314	nsP2	0,010	A>G	Transition	Lys	Synonymous
WT_ISA #1	3947	nsP2	0,013	G>A	Transition	Val > Ile	Non-Synonymous
WT_ISA #1	4729	nsP3	0,027	T>C	Transition	Thr	Synonymous
WT_ISA #1	6226	nsP4	0,011	A>G	Transition	Val	Synonymous
WT_ISA #1	6589	nsP4	0,055	G>A	Transition	Lys	Synonymous
WT_ISA #1	7742	capsid	0,869	A>G	Transition	Gln > Arg	Non-Synonymous
WT_ISA #1	7980	capsid	0,012	A>G	Transition	Ala	Synonymous
WT_ISA #1	8192	capsid	0,014	A>G	Transition	Lys > Arg	Non-Synonymous
WT_ISA #1	8824	E2	0,013	G>A	Transition	Gly > Arg	Non-Synonymous
WT_ISA #1	9175	E2	0,089	A>G	Transition	Thr > Ala	Non-Synonymous
WT_ISA #1	10948	E1	0,012	A>G	Transition	Lys > Glu	Non-Synonymous
WT_ISA #2	75	5'NTR	0,014	T>C	Transition		
WT_ISA #2	1039	nsP1	0,092	T>C	Transition	Val	Synonymous
WT_ISA #2	943	nsP1	0,087	A>G	Transition	Arg	Synonymous
WT_ISA #2	1849	nsP2	0,019	A>G	Transition	Arg	Synonymous
WT_ISA #2	2448	nsP2	0,015	C>T	Transition	Ala > Val	Non-Synonymous
WT_ISA #2	2484	nsP2	0,016	T>C	Transition	Leu > Ser	Non-Synonymous
WT_ISA #2	3674	nsP2	0,094	A>G	Transition	Thr > Ala	Non-Synonymous
WT_ISA #2	3853	nsP2	0,102	C>T	Transition	Ile	Synonymous
WT_ISA #2	3916	nsP2	0,636	T>C	Transition	Phe	Synonymous
WT_ISA #2	4030	nsP2	0,143	T>C	Transition	Asn	Synonymous
WT_ISA #2	5151	nsP3	0,102	A>G	Transition	Asp > Gly	Non-Synonymous
WT_ISA #2	5172	nsP3	0,106	C>T	Transition	Ala > Val	Non-Synonymous
WT_ISA #2	5530	nsP3	0,626	A>G	Transition	Gly	Synonymous
WT_ISA #2	5569	nsP3	0,127	A>G	Transition	Gly	Synonymous
WT_ISA #2	5911	nsP4	0,090	A>G	Transition	Arg	Synonymous
WT_ISA #2	6460	nsP4	0,014	A>G	Transition	Ala	Synonymous
WT_ISA #2	6637	nsP4	0,099	T>C	Transition	Cys	Synonymous
WT_ISA #2	7555	liaison	0,096	T>C	Transition		
WT_ISA #2	7676	capsid	0,132	A>G	Transition	Gln > Arg	Non-Synonymous

WT_ISA #2	7771	capsid	0,635	A>G	Transition	Lys > Glu	Non-Synonymous
WT_ISA #2	9209	E2	0,016	A>G	Transition	Asp > Gly	Non-Synonymous
WT_ISA #2	9381	E2	0,162	A>G	Transition	Lys	Synonymous
WT_ISA #2	10175	E1	0,096	A>G	Transition	Lys > Arg	Non-Synonymous
WT_ISA #2	10377	E1	0,119	T>C	Transition	Ser	Synonymous
WT_ISA #2	10738	E1	0,094	G>A	Transition	Ala > Thr	Non-Synonymous
WT_ISA #2	11206	E1	0,015	A>G	Transition	Thr > Ala	Non-Synonymous
WT_ISA #2	11247	E1	0,087	A>G	Transition	Gly	Synonymous
WT_ISA #2	11358	3'NTR	0,163	A>G	Transition		
WT_ISA #2	11428	3'NTR	0,161	A>G	Transition		
WT_ISA #2	11496	3'NTR	0,125	T>C	Transition		
WT_ISA #2	11761	3'NTR	0,016	T>C	Transition		
WT_ISA #3	502	nsP1	0,020	A>G	Transition	Arg	Synonymous
WT_ISA #3	833	nsP1	0,127	A>G	Transition	Lys > Glu	Non-Synonymous
WT_ISA #3	1697	nsP2	0,018	C>T	Transition	Pro > Ser	Non-Synonymous
WT_ISA #3	2298	nsP2	0,456	T>C	Transition	Val > Ala	Non-Synonymous
WT_ISA #3	3460	nsP2	0,138	A>G	Transition	Glu	Synonymous
WT_ISA #3	3609	nsP2	0,048	A>G	Transition	Asn > Ser	Non-Synonymous
WT_ISA #3	3769	nsP2	0,457	A>G	Transition	Gln	Synonymous
WT_ISA #3	4234	nsP3	0,154	A>G	Transition	Gly	Synonymous
WT_ISA #3	4828	nsP3	0,117	A>G	Transition	Ala	Synonymous
WT_ISA #3	5151	nsP3	0,444	A>G	Transition	Asp > Gly	Non-Synonymous
WT_ISA #3	5182	nsP3	0,111	A>G	Transition	Pro	Synonymous
WT_ISA #3	5199	nsP3	0,114	C>T	Transition	Ala > Val	Non-Synonymous
WT_ISA #3	5243	nsP3	0,165	T>C	Transition	Ser > Pro	Non-Synonymous
WT_ISA #3	5701	nsP4	0,139	A>G	Transition	Leu	Synonymous
WT_ISA #3	6872	nsP4	0,020	T>C	Transition	Leu	Synonymous
WT_ISA #3	7097	nsP4	0,045	T>C	Transition	Leu	Synonymous
WT_ISA #3	7111	nsP4	0,016	A>G	Transition	Arg	Synonymous
WT_ISA #3	7297	nsP4	0,130	A>G	Transition	Glu	Synonymous
WT_ISA #3	7778	capsid	0,156	A>G	Transition	Lys > Arg	Non-Synonymous
WT_ISA #3	7815	capsid	0,163	A>G	Transition	Gln	Synonymous
WT_ISA #3	8081	capsid	0,449	A>G	Transition	Lys > Arg	Non-Synonymous
WT_ISA #3	8391	E3	0,419	G>A	Transition	Thr	Synonymous
WT_ISA #3	8655	E2	0,404	A>G	Transition	Arg	Synonymous
WT_ISA #3	8922	E2	0,039	C>T	Transition	His	Synonymous
WT_ISA #3	9106	E2	0,054	A>G	Transition	Lys > Glu	Non-Synonymous
WT_ISA #3	9919	6k peptide	0,067	A>G	Transition	Arg > Gly	Non-Synonymous
WT_ISA #3	10059	E1	0,157	T>C	Transition	Pro	Synonymous
WT_ISA #3	10322	E1	0,126	A>G	Transition	Lys > Arg	Non-Synonymous
WT_ISA #3	10458	E1	0,154	A>G	Transition	Thr	Synonymous
WT_ISA #3	10473	E1	0,136	A>G	Transition	Lys	Synonymous
WT_ISA #3	10518	E1	0,157	C>T	Transition	Asn	Synonymous
WT_ISA #3	10524	E1	0,014	T>C	Transition	Ile	Synonymous
WT_ISA #3	11482	3'NTR	0,029	A>G	Transition		
WT_ISA #3	11528	3'NTR	0,462	A>G	Transition		
ΔWT_IC #1 P1	5467	nsP3	0,805	G>A	Transition	Pro	Synonymous
ΔWT_IC #1 P1	8467	E3	0,012	G>A	Transition	Asp > Asn	Non-Synonymous
ΔWT_IC #1 P4	5467	nsP3	0,872	G>A	Transition	Pro	Synonymous
ΔWT_IC #1 P4	10509	E1	0,011	C>T	Transition	Pro	Synonymous
ΔWT_IC #1 P4	10542	E1	0,014	C>T	Transition	Asp	Synonymous
ΔWT_IC #2 P1	1597	nsP1	0,997	C>T	Transition	Arg	Synonymous

ΔWT_IC #2 P4	588	nsP1	0,012	G>A	Transition	Arg > Glu	Non-Synonymous
ΔWT_IC #2 P4	1597	nsP1	0,997	C>T	Transition	Arg	Synonymous
ΔWT_IC #2 P4	2077	nsP2	0,011	C>T	Transition	Tyr	Synonymous
ΔWT_IC #2 P4	5307	nsP3	0,018	C>T	Transition	Thr > Ile	Non-Synonymous
ΔWT_IC #2 P4	7189	nsP4	0,010	T>C	Transition	Gly	Synonymous
ΔWT_ISA #1 P1	3068	nsP2	0,404	A>G	Transition	Ser > Gly	Non-Synonymous
ΔWT_ISA #1 P1	4309	nsP3	0,291	G>A	Transition	Glu	Synonymous
ΔWT_ISA #1 P1	4648	nsP3	0,050	C>T	Transition	Ser	Synonymous
ΔWT_ISA #1 P1	5110	nsP3	0,274	C>T	Transition	Phe	Synonymous
ΔWT_ISA #1 P1	5600	nsP3	0,287	A>G	Transition	Thr > Ala	Non-Synonymous
ΔWT_ISA #1 P1	7938	capsid	0,072	A>C	Transversion	Thr	Synonymous
ΔWT_ISA #1 P1	8643	E2	0,029	A>G	Transition	Leu	Synonymous
ΔWT_ISA #1 P1	9869	6k peptide	0,022	T>C	Transition	Leu > Ser	Non-Synonymous
ΔWT_ISA #1 P1	10339	E1	0,029	A>G	Transition	Thr > Ala	Non-Synonymous
ΔWT_ISA #1 P4	3068	nsP2	0,250	A>G	Transition	Ser > Gly	Non-Synonymous
ΔWT_ISA #1 P4	4156	nsP3	0,707	G>A	Transition	Gly	Synonymous
ΔWT_ISA #1 P4	4309	nsP3	0,163	G>A	Transition	Glu	Synonymous
ΔWT_ISA #1 P4	4648	nsP3	0,028	C>T	Transition	Ser	Synonymous
ΔWT_ISA #1 P4	4903	nsP3	0,058	T>C	Transition	Leu	Synonymous
ΔWT_ISA #1 P4	5110	nsP3	0,153	C>T	Transition	Phe	Synonymous
ΔWT_ISA #1 P4	5301	nsP3	0,720	A>T	Transversion	Asn > Ile	Non-Synonymous
ΔWT_ISA #1 P4	5600	nsP3	0,164	A>G	Transition	Thr > Ala	Non-Synonymous
ΔWT_ISA #1 P4	10509	E1	0,013	C>T	Transition	Pro	Synonymous
ΔWT_ISA #2 P1	5073	nsP3	0,099	A>G	Transition	Glu > Gly	Non-Synonymous
ΔWT_ISA #2 P1	5713	nsP4	0,105	A>G	Transition	Ser	Synonymous
ΔWT_ISA #2 P1	5971	nsP4	0,575	T>C	Transition	Tyr	Synonymous
ΔWT_ISA #2 P1	8620	E2	0,084	T>C	Transition	Ser > Pro	Non-Synonymous
ΔWT_ISA #2 P1	9180	E2	0,447	A>G	Transition	Thr	Synonymous
ΔWT_ISA #2 P4	5073	nsP3	0,078	A>G	Transition	Glu > Gly	Non-Synonymous
ΔWT_ISA #2 P4	5340	nsP3	0,012	C>A	Transversion	Thr > Lys	Non-Synonymous
ΔWT_ISA #2 P4	5713	nsP4	0,074	A>G	Transition	Ser	Synonymous
ΔWT_ISA #2 P4	5971	nsP4	0,578	T>C	Transition	Tyr	Synonymous
ΔWT_ISA #2 P4	6761	nsP4	0,093	A>G	Transition	Thr > Ala	Non-Synonymous
ΔWT_ISA #2 P4	8620	E2	0,159	T>C	Transition	Ser > Pro	Non-Synonymous
